# Global Trends in the Burden of Ischemic Stroke Attributable to Diabetes Versus Atrial Fibrillation

**DOI:** 10.1161/STROKEAHA.125.054141

**Published:** 2026-07-17

**Authors:** Liuding Wang, Fa Lin, Yifan Chen, Xiao Liang, Li Ma, Tzak Sing Lau, Lelin Zhang, Runting Li, Yi Liu, James Tin Fong Zhuang, Yanming Xie, Yunling Zhang, Mario Teo, Zixiao Li, Walavan Sivakumar, Xiaolin Chen, Jian Lyu

**Affiliations:** Xiyuan Hospital, China Academy of Chinese Medical Sciences, Beijing (L.W., Y.C., X.L., Y.Z., J.L.).; Department of Neurosurgery (F.L., R.L., X.C.), Beijing Tiantan Hospital, Capital Medical University, China.; Department of Neurology (Z.L.), Beijing Tiantan Hospital, Capital Medical University, China.; Pacific Neuroscience Institute and Saint John’s Cancer Institute, Providence Saint John’s Health Center, Santa Monica, CA (W.S.).; Department of Neurosurgery, Bristol Institute of Clinical Neuroscience, Southmead Hospital, North Bristol NHS Trust, United Kingdom (M.T.).; Department of Neurological Surgery, University of Pittsburgh Medical Center, University of Pittsburgh School of Medicine, PA (L.M.).; Department of Neurosurgery, Beth Israel Deaconess Medical Center, Harvard Medical School (T.S.L.).; University of Wisconsin-Madison 5228 (L.Z.).; Department of Neurology (L.W., X.L., Y.Z., J.L.), Xiyuan Hospital, China Academy of Chinese Medical Sciences, Beijing.; Department of Cardiology (Y.C.), Xiyuan Hospital, China Academy of Chinese Medical Sciences, Beijing.; NMPA Key Laboratory for Clinical Research and Evaluation of Traditional Chinese Medicine, Beijing (J.L.).; China National Clinical Research Center for Chinese Medicine Cardiology, Beijing (J.L.).; China National Clinical Research Center for Neurological Diseases, Beijing (F.L., R.L., X.C.).; Institute of Basic Research in Clinical Medicine, China Academy of Chinese Medical Sciences, Beijing (Y.L., Y.X.).; Department of Surgery, School of Clinical Medicine, Li Ka Shing Faculty of Medicine, The University of Hong Kong, Queen Mary Hospital, China (J.T.F.Z.).

**Keywords:** atrial fibrillation, body mass index, diabetes, global health, incidence, ischemic stroke

## Abstract

**BACKGROUND:**

Ischemic stroke (IS) related to atrial fibrillation (AF) represents a cardio-cerebral comorbidity, whereas diabetes-related IS reflects an advanced manifestation of panvascular disease. We aimed to analyze the burden trends in AF-related and diabetes-related IS, using Global Burden of Diseases 2021 data.

**METHODS:**

To quantify the burdens of AF- and diabetes-related IS, we applied the population-attributable fraction, the proportion of burden preventable by eliminating a risk factor. We calculated population-attributable fractions stratified by time, location, and age group. We analyzed the burdens using joinpoint regression, time-series projection to 2050, and age/sex-stratified.

**RESULTS:**

Globally, AF-related IS had a higher age-standardized incidence rate in 1990 but declined steadily (average annual percentage change =–0.81 [95% CI, –0.92 to –0.69]). Conversely, diabetes-related IS, despite a lower initial incidence, exhibited substantial and sustained increases (average annual percentage change=1.10 [95% CI, 1.02–1.18]). Middle-high socio-demographic index regions experienced the greatest burden from both comorbidities. In North America and Eastern Europe, the age-standardized incidence rates of AF-related IS stabilized (average annual percentage change=–1.45 [95% CI, –1.63 to –1.27]; –0.95 [95% CI, –1.06 to –0.84]), whereas the age-standardized incidence rates of diabetes-related IS continued to increase (average annual percentage change=1.18 [95% CI, 1.02–1.34]; 0.72 [95% CI, 0.56–0.88]). High body mass index emerged as a critical shared risk factor for AF, diabetes, and IS. Projections indicate a substantial increase in diabetes-related IS incidence over the next 3 decades, contrasting with minimal growth in AF-related IS.

**CONCLUSIONS:**

IS remains a critical global health challenge, primarily driven by the rising burden of diabetes-related IS. Our findings highlight the importance of age- and country-specific interventions.

Ischemic stroke (IS) remains a major global cause of mortality and disability.^1^ With accelerating population aging, the frequent coexistence of IS with other chronic conditions has gained increasing global attention.^2^ Among these, diabetes and atrial fibrillation (AF) constitute 2 major independent risk factors for IS.^3,4^ In 2021, ≈529 million people worldwide had diabetes,^5^ while AF and atrial flutter affected around 52.6 million individuals globally.^6^ Pathophysiologically, diabetes contributes to IS by promoting chronic inflammation, endothelial dysfunction, and thrombus formation within atherosclerotic cerebral arteries, predominantly leading to thrombotic stroke. In contrast, AF predisposes individuals to IS by promoting cardiac thrombus formation and subsequent embolization, typically leading to embolic strokes. From panvascular and cardio-cerebral perspectives, diabetes-related and AF-related IS represent 2 clinically important patterns of multimorbidity.^7,8^ Diabetes frequently coexists with IS; for instance, a large-scale study from China involving 838 229 patients with IS reported a diabetes prevalence of ≈34.2%.^9^ Similarly, AF commonly coexists with IS, affecting ≈20% to 30% of patients with IS.^10^ Both comorbidities are significantly associated with increased mortality and adverse events during hospitalization.^9,11^

However, existing global research addressing these 2 prevalent comorbidity patterns remains insufficient in several important aspects. First, most epidemiological studies have assessed the disease burdens of diabetes, AF, or IS independently, without explicitly addressing their co-occurrence.^1,5,6^ Secondly, existing research has primarily focused on disease-specific risk factors, often neglecting the risk factors common to both comorbidities. Crucially, systematic comparisons addressing global geographic variations and longitudinal trends between these 2 predominantly unidirectional comorbidity patterns remain limited. To capture and compare the contributions of each comorbidity to disease burden across different populations and settings, we employed an established epidemiological metric, the population-attributable fraction (PAF). The PAF represents the proportion of disease burden in a population that would be prevented if a given risk factor were eliminated, assuming a causal relationship. We introduced the PAF to quantify the burden of IS attributable to comorbidities with a defined causal link to it, allowing a direct comparison of the burden related to diabetes versus that related to AF.

Therefore, this study employs data from the GBD (Global Burden of Diseases, Injuries, and Risk Factors Study) 2021 to systematically compare the burden of IS related to diabetes versus that related to AF. By quantifying age- and region-specific burden trends for these 2 risk factors, we sought to identify high-risk subgroups. Our findings will inform evidence-based strategies to facilitate tailored regional IS prevention, optimize global stroke resource allocation, and ultimately reduce the global disease burden.

## Methods

All data are publicly available on the GBD website. As no patient-specific data were used, informed consent was not required.

### Overview

This study is reported following the Guidelines for Accurate and Transparent Health Estimates Reporting, applicable to global health estimates.^12^ Data for this study were sourced from the GBD 1990 to 2021, published in 2024.^13^ We retrieved relevant burden data for IS, diabetes, and AF from 204 countries and territories covering the period 1990 to 2021, using the Global Health Data Exchange online database (http://ghdx.healthdata.org/gbd-results-tool). The GBD calculates disease prevalence and incidence using a Bayesian meta-regression model (DisMod-MR 2.1), estimates mortality rates through the Cause of Death Ensemble Model, and computes disability-adjusted life-years (DALYs) by summing years lived with disability and years of life lost.^13^ The GBD uses a Bayesian probabilistic framework to estimate uncertainty as a probability distribution. Probability reflects uncertainty about observed or hypothetical events under given assumptions, and the likely range of the true value is estimated via multiple sampling simulations.

### Population Attributable Fraction of IS

We estimated the population attributable fractions (PAFs) separately for IS burden related to diabetes and AF, using the Levin formula: $PAF=\frac{p\times \left(RR-1\right)}{p\times \left(RR-1\right)+1}$.^14–16^ The variable relative risk (RR) represents the RR of IS among exposed versus unexposed individuals, and the variable p is the prevalence of the exposure in the general population across different regions, time periods, and age groups. Diabetes and AF prevalence estimates were obtained directly from the GBD database. For estimating the PAF of IS burden related to diabetes, an RR estimate was derived from a review of relevant literature. A case-control study involving 26 919 participants (including 10 388 IS cases) from 32 countries reported that individuals with diabetes or HbA1c levels ≥6.50% had a 1.33-fold increased risk of IS (95% CI, 1.18–1.50) compared with those without diabetes and with HbA1c <6.5%.^17^ For estimating the PAF of IS burden related to AF, we adopted a RR of 4.59 (95% CI, 3.66–5.75).^17^ The PAF estimates are specific to time, geographic location, and age group (Tables S1 and S2).

The global incidence of IS is ≈100 per 100 000,^1^ which represents a low-incidence event. Under such conditions, the odds ratio (OR) derived from the case-control study can be reasonably interpreted as an approximation of the RR. Since our study calculated the PAF using this value, we further conducted a sensitivity analysis to quantify the uncertainty arising from approximating RR using OR, as well as the uncertainty in prevalence from the GBD, and to assess the robustness of our findings. Specifically, based on the 95% CI of the OR, we performed Monte Carlo simulations to estimate the plausible range of the PAF (Tables S1 and S2).

It should be noted that the PAF-based estimates presented in this study are derived under a standardized modeling framework, which assumes a standardized OR that is constant across the study period and consistent across different populations globally. Consequently, these estimates should be interpreted as approximate comparative burden measures rather than precise causal effect sizes.

### Global Burden of IS Related to Diabetes or AF

We applied an age-stratified attribution method to estimate the IS burden related to diabetes or AF by region and year. Specifically, the population was stratified into 5-year age bands ranging from 5 years to 95 years and older; PAFs were calculated for each age group and applied to corresponding IS incidence, DALYs, and mortality rates, and subsequently aggregated across all age groups to derive total IS incidence, DALYs, and deaths related to diabetes or AF for each region and year. This approach preserves age-specific characteristics of the disease burden while accurately reflecting the overall population burden through aggregation. Additionally, to minimize the influence of variability in population age structures, we calculated age-standardized incidence rates (ASIRs), age-standardized mortality rates (ASMRs), and age-standardized DALY rates (ASDRs) for each age group, using the GBD world standard population.

### Average Annual Percent Change

Joinpoint regression analysis (version 5.1.0.0) was employed to calculate the average annual percentage change (AAPC) in ASIR, ASMR, and ASDR from 1990 to 2021, along with corresponding 95% CIs. The Grid Search method was used to evaluate all potential joinpoints, selecting the joinpoint yielding the lowest mean squared error. Subsequently, a Monte Carlo permutation test was conducted to identify the optimal number of joinpoints. The final model calculated the AAPCs globally and for each country and region to quantify temporal trends from 1990 to 2021.

### Socio-Demographic Index

The socio-demographic index (SDI) is a composite indicator of development.^13^ Based on SDI values, the 204 countries and territories were categorized into 5 development levels: low, low-middle, middle, high-middle, and high SDI. SDI values range from 0 to 1, with higher values representing higher socio-economic development levels. This classification facilitates systematic analyses of the impact of socio-economic development on disease burden.

### Risk Factors

Based on GBD 2021, we identified 4 shared modifiable risk factors for AF, diabetes, and IS: dietary risks, high alcohol use, high body mass index (BMI), and smoking. Using established GBD estimation methods, we modeled exposure levels for each risk factor, identified their theoretical minimum risk thresholds, and derived PAFs. These PAFs, stratified by key demographics, were then applied to related DALYs and deaths to estimate the burden of AF, diabetes, and IS related to each factor.

### Autoregressive Integrated Moving Average Model

This study employed an Autoregressive Integrated Moving Average (ARIMA) model to forecast the global burden of diabetes- or AF- related IS over the next 30 years, based on historical data for ASIR, ASMR, and ASDR from 1990 to 2021.^18^ The ARIMA model is a time-series analytical tool designed to forecast future trends based on historical data and current patterns. The ARIMA model comprises 3 parameters: p (autoregressive component), d (differencing component), and q (moving average component). The optimal ARIMA specifications were selected using the Akaike and Bayesian information criteria. For AF-related IS, the selected models were ARIMA (0,2,1) for ASIR, ARIMA (1,1,0) for ASMR, and ARIMA (1,1,0) for ASDR. For diabetes-related IS, the corresponding models were ARIMA (0,2,1) for ASIR, ARIMA (2,0,0) for ASMR, and ARIMA (1,1,0) for ASDR. Projections assumed stable trends in risk factor exposure, population structure, and no major policy or technological disruptions. These assumptions and model selections were applied consistently to ensure comparability of projected estimates.

### Role of the Funding Source

The funder of the study had no role in the study design, data collection, data analysis, data interpretation, or writing of the report.

## Results

### Global Burden of AF-Related and Diabetes-Related IS

In 2021, 973 149 new AF-related IS cases occurred globally, a 109% increase from 1990. After adjusting for population growth and aging, the ASIR declined from 14.97 to 12.11 per 100 000 between 1990 and 2021 (AAPC=–0.81 [95% CI, –0.92 to –0.69]). ASMR and ASDR showed similar decreases, with AAPCs of –1.86 (95% CI, –1.94 to –1.77) and –1.67 (95% CI, –1.76 to –1.59), respectively (Table S3). For diabetes-related IS, 462 507 cases occurred globally in 2021, a 247.78% increase from 1990. The ASIR rose from 3.75 to 5.52 per 100 000 during this period (AAPC=1.10 [95% CI, 1.02–1.18]). Meanwhile, ASMR remained stable (AAPC=–0.02 [95% CI, –0.14 to 0.11]), whereas ASDR showed a slight increase (AAPC=0.17 [95% CI, 0.05–0.29]; Table S3). In summary, AF-related IS exhibited a higher baseline burden with a global downward trend, while diabetes-related IS began at a lower level but continued to rise worldwide.

**Figure 1.**
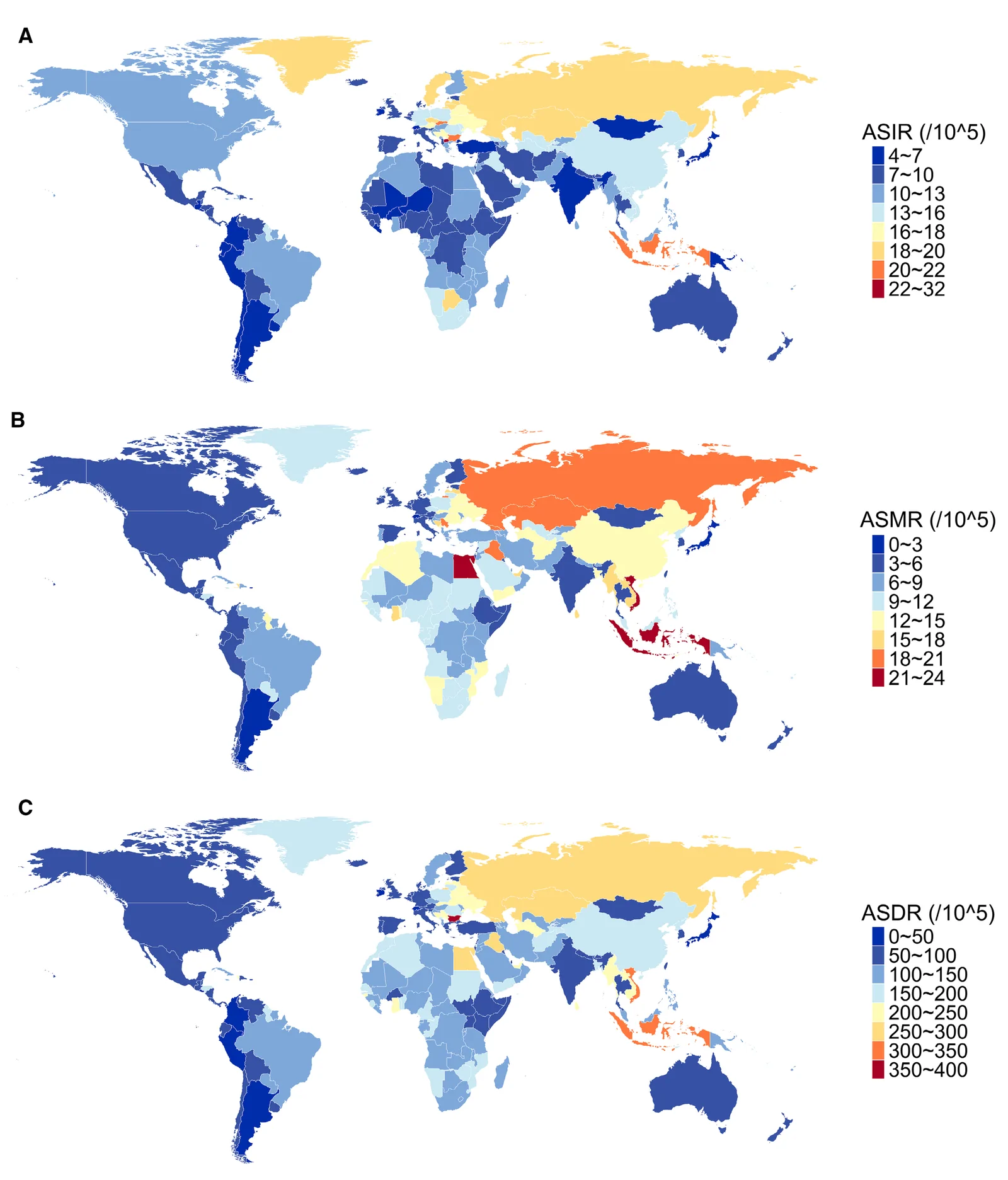
**Global burden of ischemic stroke attributable to atrial fibrillation in 2021.** Age-standardized incidence (**A**), mortality (**B**), and disability-adjusted life year (**C**) rates of ischemic stroke attributable to atrial fibrillation across 204 countries and territories in 2021. ASDR indicates age-standardized disability-adjusted life-year rate; ASIR, age-standardized incidence rate; and ASMR, age-standardized mortality rate.

**Figure 2.**
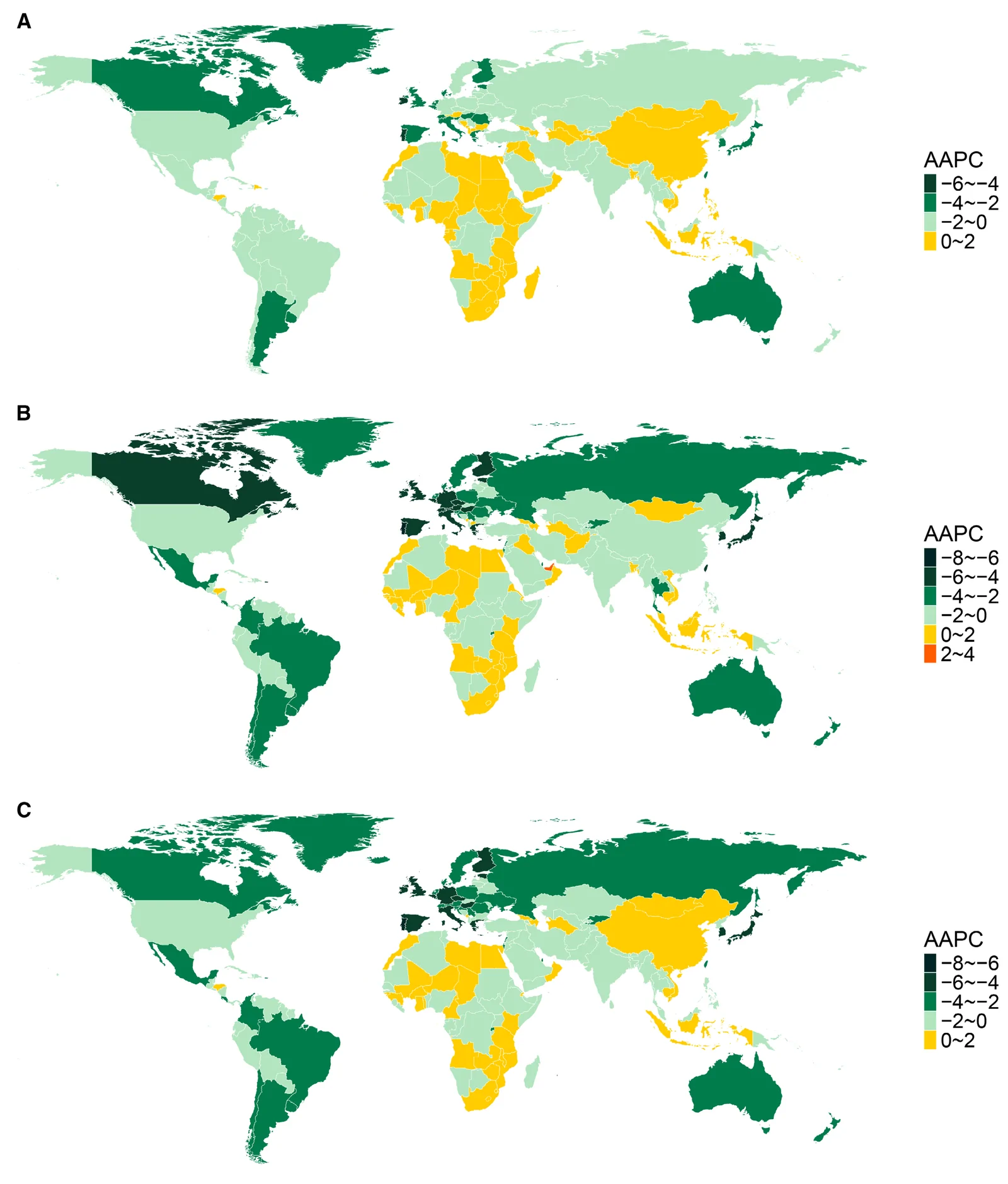
**Temporal trends in atrial fibrillation–related ischemic stroke burden, 1990–2021.** Average annual percentage changes (AAPC) in age-standardized incidence (**A**), mortality (**B**), and disability-adjusted life year (**C**) rates of atrial fibrillation-related ischemic stroke across 204 countries and territories between 1990 and 2021.

Regionally, Eastern Europe showed the highest ASIR of AF-related IS at 18.46 per 100 000, followed by Central Europe at 16.31 per 100 000 (Table S3). Countries with notably high national ASIRs included Bulgaria, Slovakia, the Czech Republic, Russia, Sweden, Latvia, and Estonia (Figure 1). In East Asia and Central Asia, high-ASIR countries included China, North Korea, Uzbekistan, and Turkmenistan (Figure 1). In contrast, the lowest ASIRs were observed in Southern Latin America (5.25 per 100 000) and Andean Latin America (6.12 per 100 000; Table S3). From 1990 to 2021, ASIR increased across East Asia, Central Asia, Southeast Asia, and much of Sub-Saharan Africa (Table S3), with China and Mongolia as representative countries (Figure 2). The high-income Asia Pacific region showed the largest decline in ASIR (AAPC=–3.33 [95% CI, –3.71 to –2.95]), with Japan and South Korea as representative countries (Table S3; Figure 2). Both Europe (AAPC=–1.89 [95% CI, –1.99 to –1.78]) and North America (AAPC=–1.45 [95% CI, –1.63 to –1.27]) showed declining ASIR trends. Notably, Eastern Europe, despite having the highest ASIR, also exhibited a decline (AAPC=−0.95 [95% CI, –1.06 to –0.84]). ASMR and ASDR patterns further highlighted regional disparities: in 2021, Eastern Europe, Central Europe, and Asian regions bore notably high burdens, whereas Latin America generally showed lower rates. The high-income Asia Pacific region recorded the lowest global ASMR and ASDR. From 1990 to 2021, increases in ASMR and ASDR were observed only in Southern Sub-Saharan Africa, Southeast Asia, and East Asia, with representative countries including South Africa, Zimbabwe, Mozambique, Zambia, and Angola (Figure 2).

**Figure 3.**
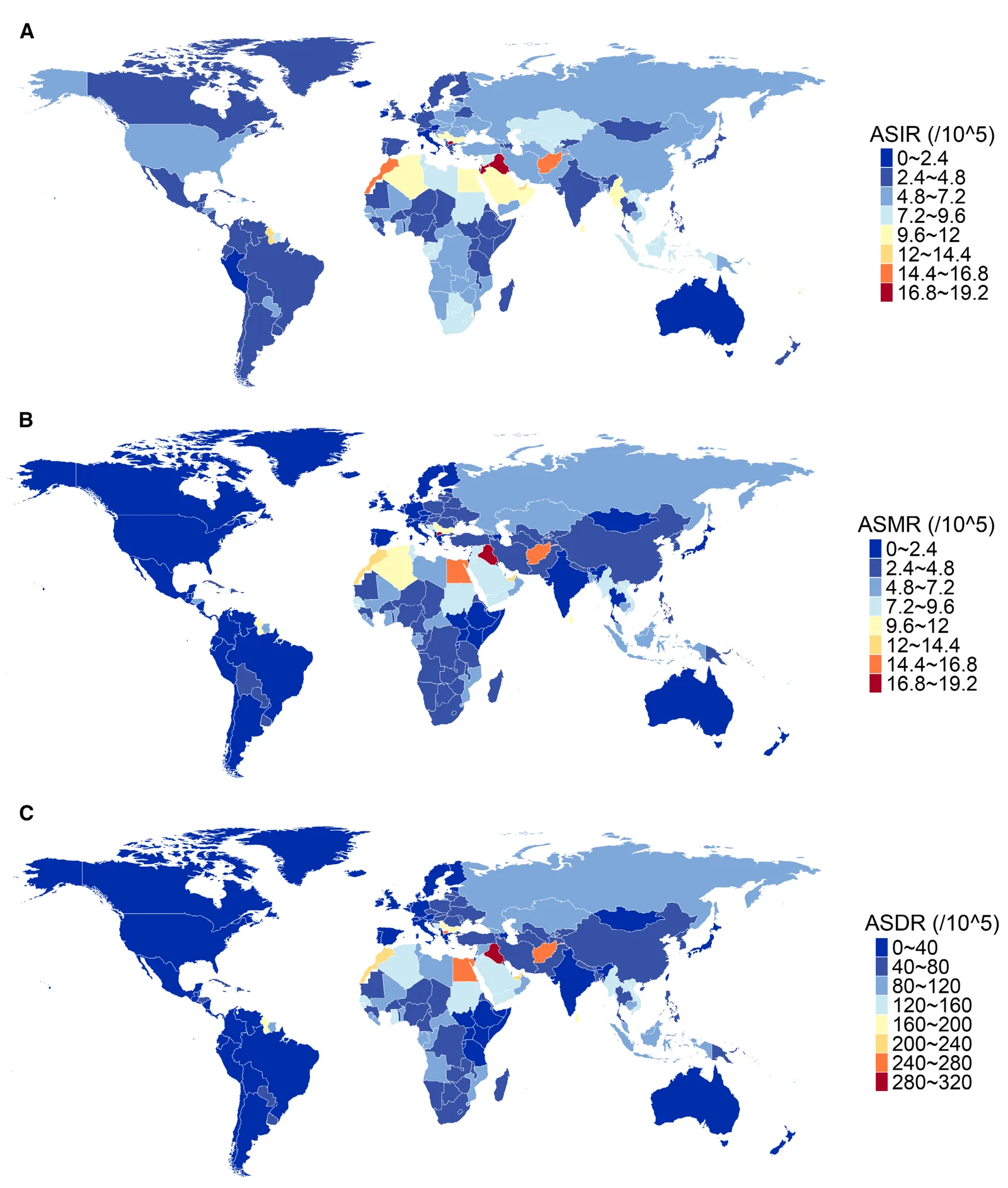
**Global burden of ischemic stroke attributable to diabetes in 2021.** Age-standardized incidence (**A**), mortality (**B**), and disability-adjusted life year (**C**) rates of ischemic stroke related to diabetes across 204 countries and territories in 2021. ASDR indicates age-standardized disability-adjusted life-year rate; ASIR, age-standardized incidence rate; and ASMR, age-standardized mortality rate.

**Figure 4.**
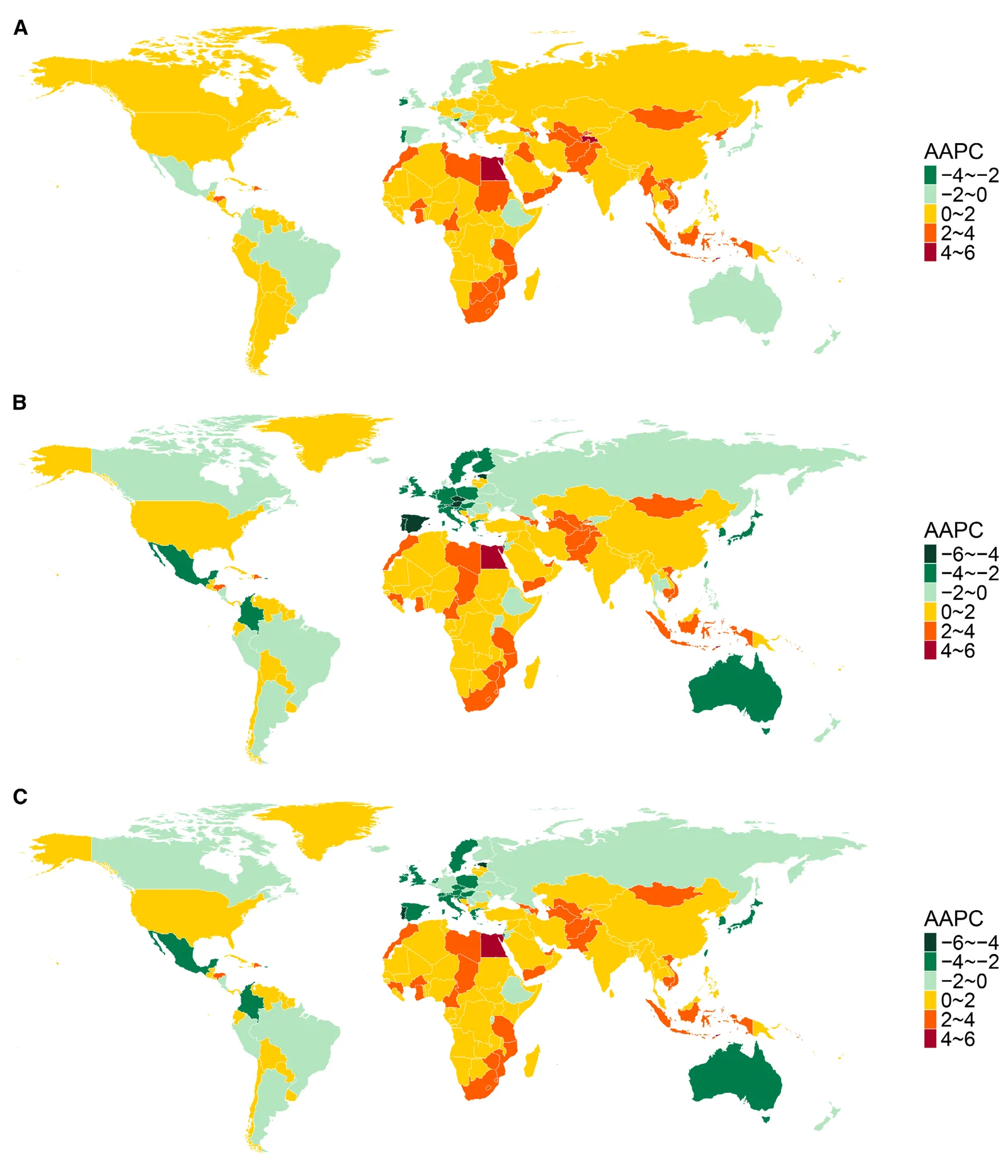
**Temporal trends in diabetes-related ischemic stroke burden, 1990–2021.** Average annual percentage changes (AAPC) in age-standardized incidence (**A**), mortality (**B**), and disability-adjusted life year (**C**) rates of diabetes-related ischemic stroke across 204 countries and territories between 1990 and 2021.

Regarding diabetes-related IS, North Africa and the Middle East had the highest ASIR at 9.08 per 100 000 (Table S3), with elevated national rates in Iraq, Jordan, and Morocco (Figure 3). This was followed by Southern Sub-Saharan Africa (8.11 per 100 000) and Oceania (7.25 per 100 000), as well as East Asia, Southeast Asia, and Central Asia, with China, Myanmar, and Afghanistan as representative countries (Table S3; Figure 3). Australasia recorded the lowest ASIR at 2.33 per 100 000. From 1990 to 2021, Asia and Africa demonstrated the most significant increases in ASIR, with North Africa, particularly Egypt (Figure 4), exhibiting the largest increase (AAPC=3.36 [95% CI, 3.31–3.42]; Table S3). North America (AAPC=1.18 [95% CI, 1.02–1.34]) and Eastern Europe (AAPC=0.72 [95% CI, 0.56–0.88]) also showed upward trends. In contrast, Central Latin America, Tropical Latin America, Western Europe, and Australasia showed declining ASIR trends. ASMR and ASDR results further highlighted regional disparities, with Asia and Africa carrying notably higher diabetes-related IS burdens. North Africa and the Middle East had the highest ASMR and ASDR, followed by Southeast Asia. Beyond Asia and Africa, North America also showed increasing trends, with AAPCs of 0.63 (95% CI, 0.30–0.97) for ASMR and 0.90 (95% CI, 0.63–1.18) for ASDR (Table S3).

Overall, our findings show that Asia and Africa face a dual high burden of IS related to both AF and diabetes, with these burdens ranking among the highest globally and continuing to rise. Although Europe continues to carry the greatest AF-related IS burden, its regional trends are declining. In contrast, diabetes-related IS burden is broadly increasing worldwide. In addition to Asia and Africa, rising rates are now evident in North America and Eastern Europe.

### Differences in AF-Related and Diabetes-Related IS Burden Across SDI Levels, Age, and Sex

**Figure 5.**
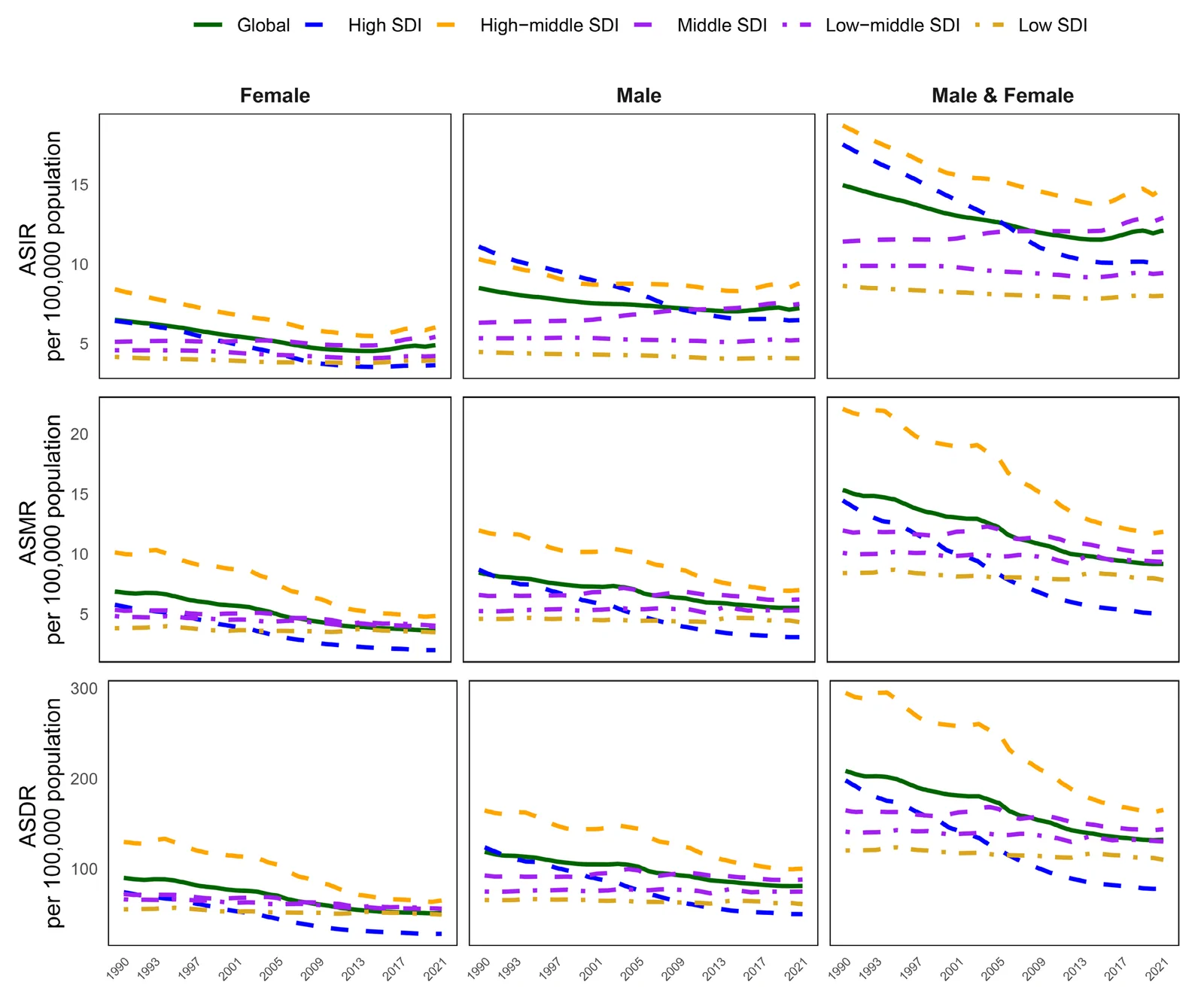
**Global and socio-demographic trends in age-standardized incidence, mortality, and disability-adjusted life year rates of atrial fibrillation-related ischemic stroke between 1990 and 2021.** ASDR indicates age-standardized disability-adjusted life-year rate; ASIR, age-standardized incidence rate; ASMR, age-standardized mortality rate; and SDI, socio-demographic index.

**Figure 6.**
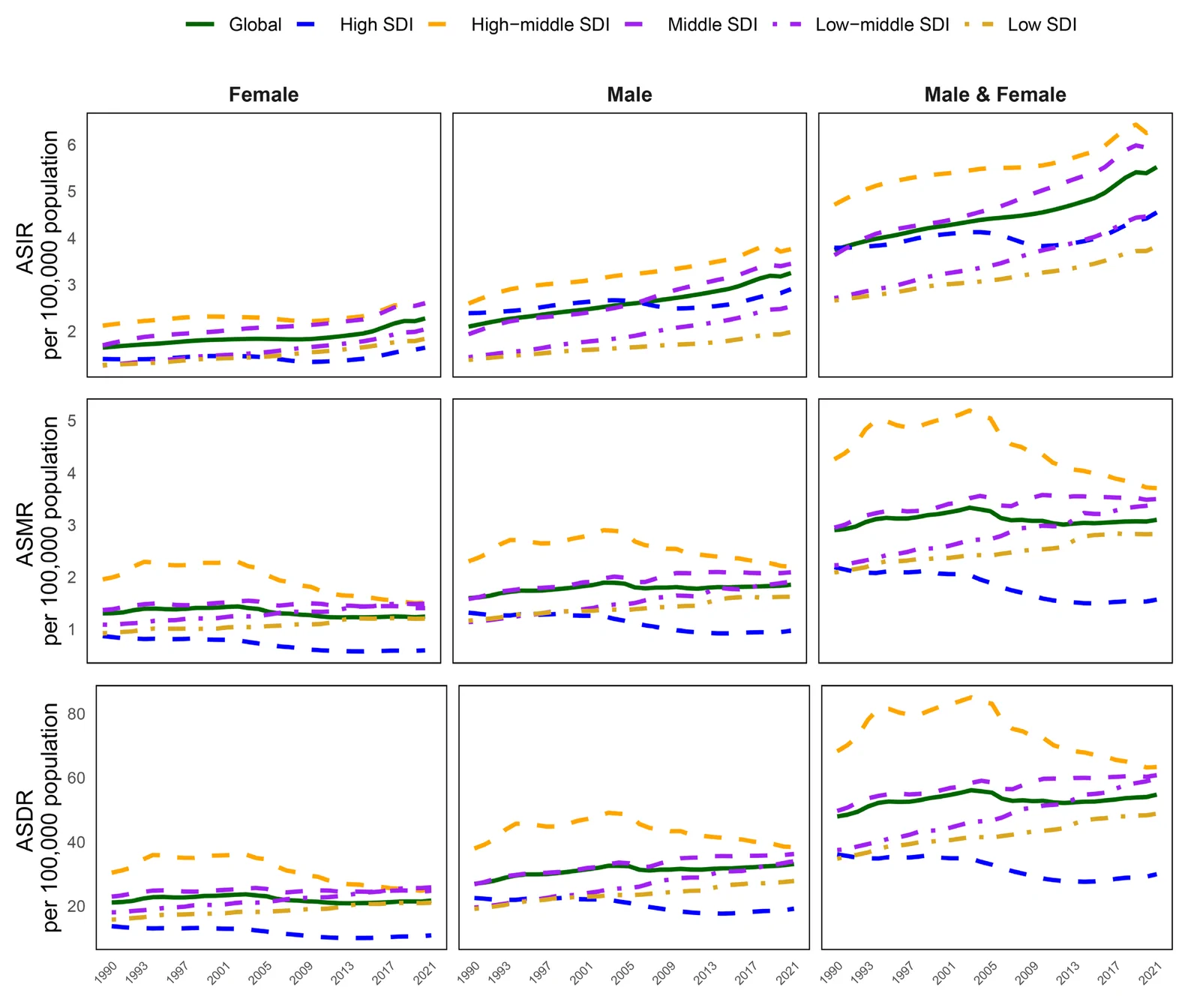
**Global and socio-demographic trends in age-standardized incidence, mortality, and disability-adjusted life year rates of diabetes-related ischemic stroke between 1990 and 2021.** ASDR indicates age-standardized disability-adjusted life-year rate; ASIR, age-standardized incidence rate; ASMR, age-standardized mortality rate; and SDI, socio-demographic index.

When stratified by SDI, high-middle SDI regions had the highest ASIR of AF-related IS in 2021 at 14.80 per 100 000, whereas low SDI regions showed the lowest rates (Table S3; Figure 5). From 1990 to 2021, only the middle SDI regions showed an increasing ASIR trend. High-middle SDI regions had the highest ASMR and ASDR, while ASMR and ASDR declined across all SDI levels, with larger reductions generally occurring in higher SDI regions. For diabetes-related IS, high-middle SDI regions again had the highest ASIR in 2021 at 6.36 per 100 000, whereas low SDI regions had the lowest rates (Table S3; Figure 6). Unlike AF, diabetes-related ASIR increased consistently across all SDI levels, with lower SDI regions showing the greatest rises. Additionally, ASMR and ASDR declined in high and high-middle SDI regions but increased in other regions, particularly in low-middle SDI regions. Overall, high-middle SDI regions bore the highest burden for both conditions.

**Figure 7.**
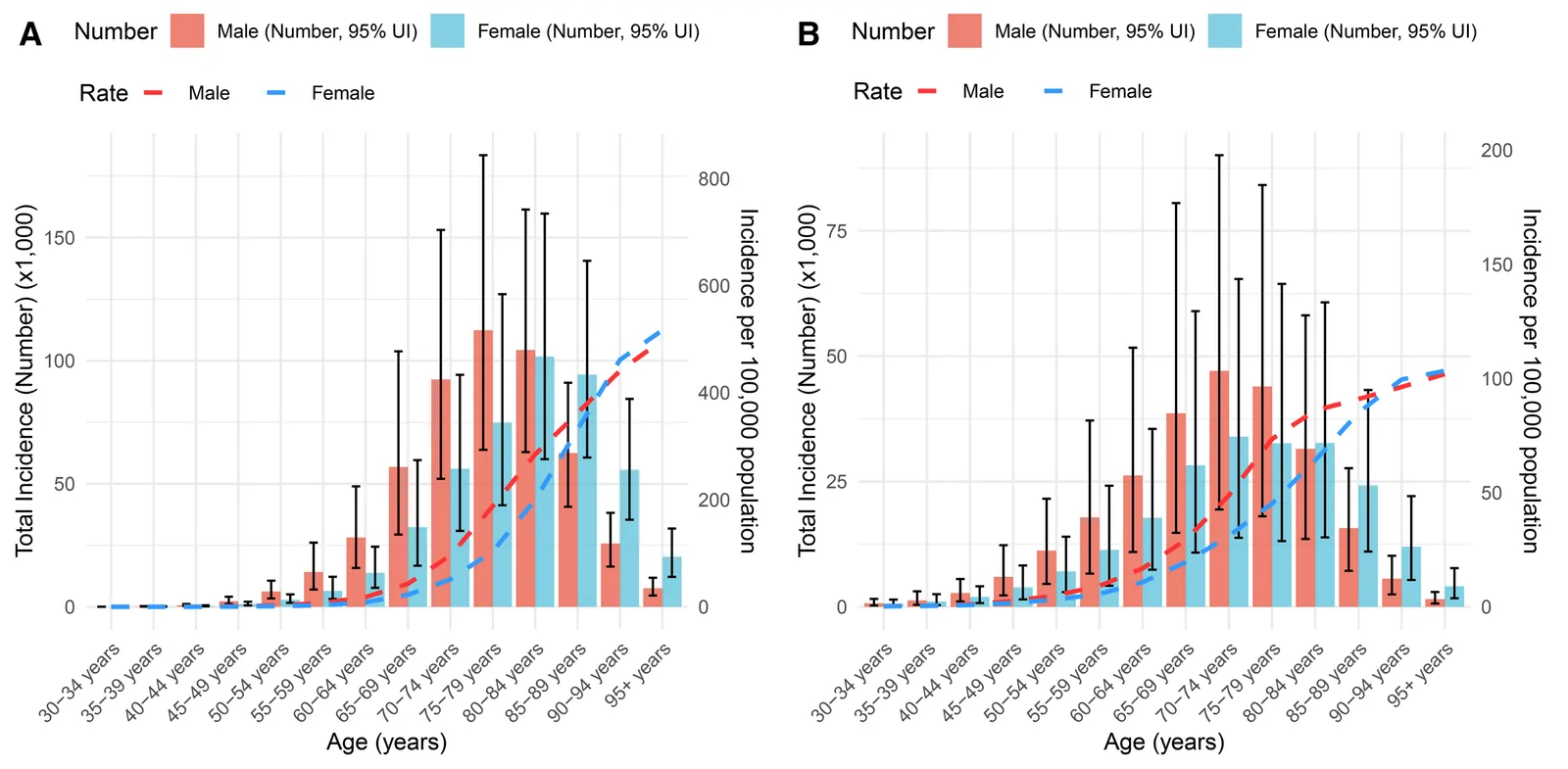
**Age- and sex-specific burden of atrial fibrillation and diabetes-related ischemic stroke in 2021.** Incidence count and age-standardized rates of atrial fibrillation (**A**) and diabetes-related ischemic stroke (**B**) by sex and age group, 2021. The error bars show the 95% uncertainty intervals for the number of cases.

Global data from 2021 showed clear age- and sex-specific differences in the epidemiological patterns of AF-related IS. Among men, the highest number of incident cases occurred at ages 75 to 79 years (112 371 cases), whereas in women, the peak was at ages 80 to 84 years (101 706 cases; Figure 7; Table S4). Mortality data showed the highest number of deaths among men aged 80 to 84 years (85 885 cases) and among women aged 85 to 89 years (89 223 cases; Table S5; Figure S1). DALY analysis indicated the greatest burden in the 80 to 84 years group, with 1 209 835 DALYs in men and 1 159 752 DALYs in women (Table S6; Figure S1). Notably, a consistent age-sex pattern was observed across incidence, mortality, and DALYs: men experienced a higher burden before age 80, burdens converged at ages 80 to 84, and women surpassed men after age 85. Further analysis of age-standardized indicators showed that incidence, mortality, and DALY rates of AF-related IS consistently increased with advancing age. Diabetes-related IS demonstrated a similar age-sex pattern to that observed for AF-related IS (Figure 7; Tables S7 through S9; Figure S2).

### Risk Factors of AF-Related and Diabetes-Related IS

High BMI, smoking, excessive alcohol consumption, and dietary risks are shared modifiable risk factors for AF, diabetes, and IS, with high BMI contributing most substantially to both AF and diabetes-related IS burden. Specifically, high BMI contributed to 8.00% of AF-related deaths and 8.70% of AF-related DALYs globally (Figure S3), and had an even greater impact on diabetes, accounting for 43.70% of diabetes-related deaths and 49.80% of diabetes-related DALYs (Figure S4). It also independently accounted for 4.80% of IS-related deaths and 6.30% of IS-related DALYs worldwide (Figure S5). Regional analysis showed that North Africa and the Middle East had the highest burden of high BMI-related disease. Furthermore, when stratified by SDI, high-SDI regions had the greatest burden attributable to high BMI.

### Global Prediction of Future AF-Related and Diabetes-Related IS Burden

**Figure 8.**
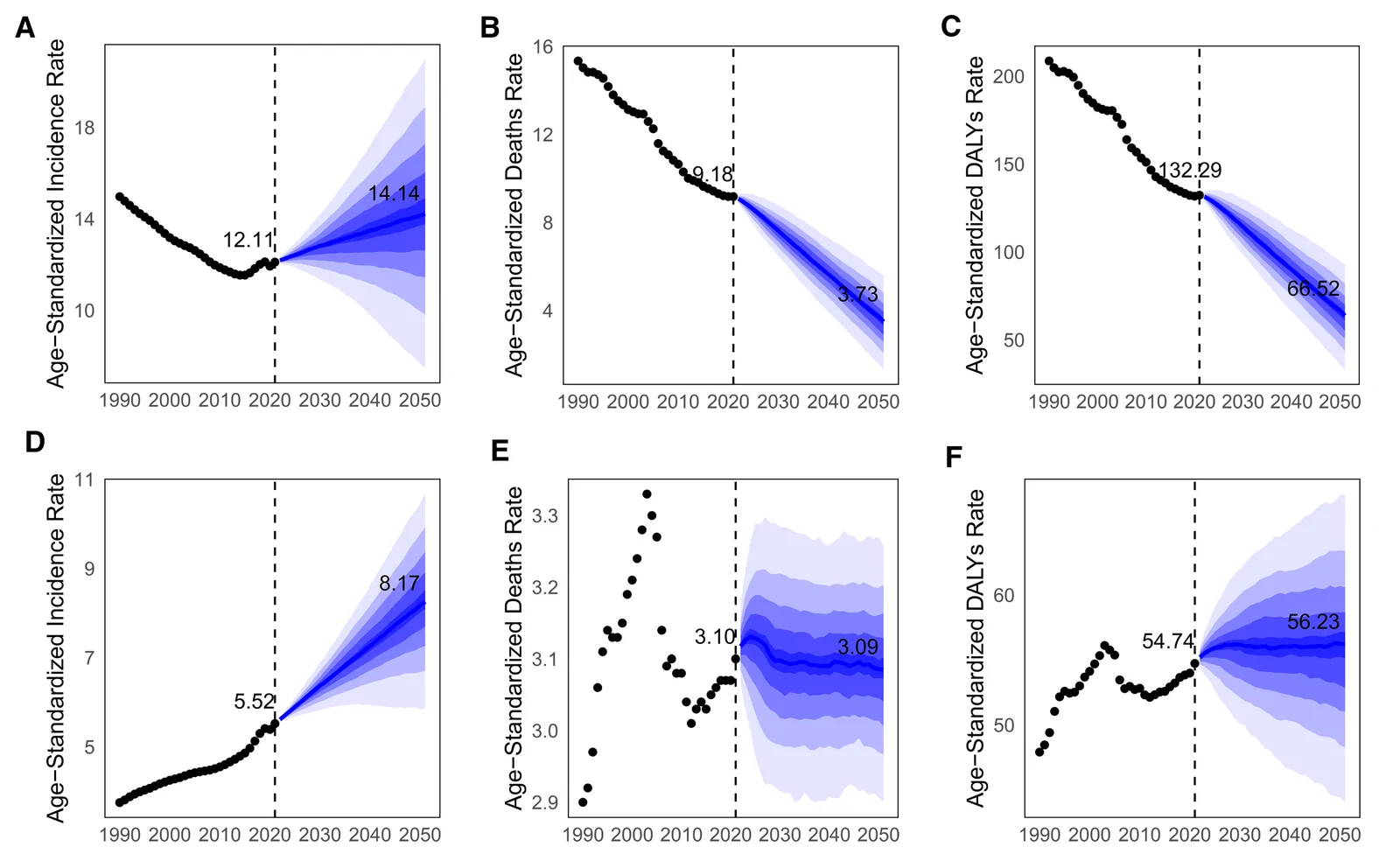
**Projected global burden of atrial fibrillation and diabetes-related ischemic stroke, 2021–2050.** Predicted trends of age-standardized incidence, mortality, and disability-adjusted life year rates for atrial fibrillation (**A–C**) and diabetes-related ischemic stroke (**D–F**) worldwide, 2021 to 2050. The colored lines are surrounded by gradient-shaded areas that represent the 95% uncertainty ranges. Wider gradients indicate greater uncertainty.

By 2050, the ASIR of AF-related IS is projected to rise from 12.11 to 14.14 per 100 000, an increase of ≈16.76% (Figure 8). The ASIR of diabetes-related IS is projected to rise by about 48%, increasing from 5.52 to 8.17 per 100 000 by 2050 (Figure 8). Over the same period, the ASMR and ASDR for AF-related IS are expected to decline markedly, from 9.18 and 132.29 per 100 000 in 2021 to 3.73 and 66.52 per 100 000 (Figure 8). In contrast, the ASMR and ASDR for diabetes-related IS are projected to remain relatively stable (Figure 8).

### Sensitivity Analysis

We conducted a probabilistic uncertainty analysis using Monte Carlo simulation (10 000 iterations), in which the ORs were randomly sampled from their uncertainty distributions to generate PAF distributions. For most estimates, the 95% uncertainty intervals were narrow, with relative widths (upper bound minus lower bound, divided by the point estimate) of ≤30%. This indicates that the PAF estimates are relatively robust to uncertainty in the underlying ORs, supporting the reliability of the estimated burdens of AF-related and diabetes-related IS.

## Discussion

Although AF and diabetes differ biologically and epidemiologically, both are clinically recognizable and prevention-relevant contributors to the global burden of IS. Fundamentally, these conditions reflect distinct pathophysiologic mechanisms and clinical management paradigms, where AF prevention centers on anticoagulation for cardioembolic risk, whereas diabetes prevention requires comprehensive control of metabolic and vascular risk factors. Unlike previous GBD studies focusing on exposure-based risks, this study adopted a condition-oriented perspective to contrast the population-level burden attributable to AF and diabetes, with the aim of defining distinct prevention priorities across regions and age groups. Using a PAF methodological framework, this study developed a global analytical model of IS burden driven by 2 key etiologically comorbid pathways: AF-related and diabetes-related IS. Our findings indicated that although AF-related IS presented a higher initial burden, it exhibited a declining global trend; by contrast, diabetes-related IS, despite its lower initial burden, continued to rise consistently across multiple regions. This pattern may reflect the more direct causal link between AF and cardioembolic stroke, whereas diabetes contributes to IS through broader and more heterogeneous vascular pathways, including atherosclerosis, small-vessel disease, and ischemic heart disease. Analysis of socioeconomic disparities revealed that high-middle SDI regions experienced the highest dual burdens of these comorbidities, whereas high SDI regions experienced the lowest burdens. Regions in Asia and Africa experienced persistently high burdens from both AF-related and diabetes-related IS. Meanwhile, North America and Eastern Europe exhibited stabilization in AF-related IS burden but continued to experience rising burdens from diabetes-related IS. Both comorbidities demonstrated characteristic age-sex crossover patterns, with males experiencing significantly higher burdens before age 80, followed by a reversal in sex disparity after age 85. High BMI, as a shared core risk factor for AF, diabetes, and IS, emerged as a critical intervention target for reducing burdens associated with these conditions. Predictive analyses indicated that diabetes-related IS incidence will substantially rise over the next 3 decades, whereas AF-related IS incidence will experience only moderate growth.

AF and diabetes have long been recognized as major risk factors for IS, but their independent contributions to the IS burden have not been systematically quantified. Based on global burden data, our study found that the ASIR of AF-attributable IS significantly exceeded that of diabetes-related IS, a finding that diverges from the typical epidemiological distribution of IS subtypes. Diabetes-related IS predominantly involves small-artery occlusion and large-artery atherosclerosis, whereas AF-related IS is primarily cardioembolic. A meta-analysis comprising 65 studies from 29 countries indicated that globally, the proportion of cardioembolic strokes is significantly lower than that of small-artery occlusion and large-artery atherosclerosis combined, with an approximate ratio of 1:2.^19^ This substantial discrepancy between attributable risks and the epidemiological distribution of subtypes underscores the multifactorial nature of IS pathogenesis, particularly for small-artery occlusion and large-artery atherosclerosis strokes. Although AF is the predominant risk factor for cardioembolic strokes, diabetes appears to have a relatively limited independent contribution to atherosclerotic strokes. Moreover, diabetes-related IS burdens, such as ASMR and ASDR, account for only 6.00% to 7.00% of the total IS burden,^1^ substantially lower than traditionally perceived theoretical contributions. In contrast, combined metabolic risk factors contribute up to 70.00% of the IS burden,^20^ highlighting the limited impact of individual risk factors and underscoring the importance of multifactorial pathogenesis. The accelerating aging population and evolving disease spectrum render comorbidity an increasingly important public health challenge. Advances in multi-omics technologies and systems epidemiology are shifting research paradigms from isolated analyses of individual risk factors towards constructing comorbidity networks and risk synergy models.^21,22^

Our study uniquely employed the PAF model, incorporating precise diabetes prevalence data to quantitatively assess the independent contribution of diagnosed diabetes to IS burden, rather than focusing solely on high fasting plasma glucose derived from the GBD database as in previous studies.^23^ High fasting plasma glucose is defined as any fasting plasma glucose concentration exceeding the theoretical minimum risk interval (4.80–5.40 mmol/L or 86.00–97.00 mg/dL).^24^ Our findings indicated that the ASMR attributable risk associated with diabetes (6.00%–7.00%) was substantially lower than the corresponding risk attributable to high fasting plasma glucose (18.36%),^25^ suggesting that populations with impaired fasting glucose contribute substantially to IS burdens, an effect previously underestimated. A cohort study in China involving 12 145 nondiabetic participants reported significantly elevated cardiovascular risks associated with prolonged prediabetic states.^26^ Similarly, a nationwide Korean cohort of 2 206 679 individuals without diabetes showed that greater cumulative exposure to impaired fasting glucose was associated with a higher risk of all-cause mortality.^27^ A large Dutch cohort further reported that impaired fasting glucose confers a substantial lifetime cardiovascular risk, particularly among men, approaching that observed in type 2 diabetes.^28^ Because prediabetic states are more prevalent than diagnosed diabetes and provide a longer window for intervention, IS prevention may need to extend further upstream into the prediabetic stage.

The burdens of AF- and diabetes-related IS mirror stark geographic inequalities in healthcare access, risk-factor control, and socioeconomic development. In parts of Asia and Africa, low rates of anticoagulation therapy for AF coexist with inadequate diabetes management, jointly exacerbating IS burdens. In China, only 60.60% of urban and 45.90% of rural AF patients receive treatment, with anticoagulation therapy uptake even lower at 22.40% and 17.20%, respectively.^29^ By contrast, Europe and the United States have established standardized AF management practices owing to more developed healthcare systems. This is consistent with a Danish study reporting a decreased lifetime risk of stroke after AF.^30^ Nevertheless, these regions continue to face an increasing burden of diabetes-related IS driven by the rising prevalence of metabolic syndrome. The sustained increase in metabolic disease burden has deep-rooted social determinants, including dietary shifts and decreased physical activity resulting from rapid urbanization. Socioeconomic development has driven a substantial rise in per capita meat consumption, which exhibits a clear positive correlation with type 2 diabetes prevalence in North America, Europe, and the Western Pacific.^31^ Our study further highlights high BMI as a shared risk factor for both AF-related and diabetes-related IS, with high SDI regions shouldering the greatest high-BMI-related disease burdens. One study reported that, over the past 30 years, global deaths and DALYs attributable to high BMI have increased > 2.50-fold among both women and men.^32^ High BMI is closely associated with modifiable behavioral risk factors, particularly dietary changes and reduced physical activity. Addressing these issues requires strategies beyond medical care improvements, emphasizing early intervention, comprehensive management of metabolic disorders, and public health policy interventions targeting social determinants, such as promoting healthy diets and implementing population-wide weight reduction initiatives.

The ASIR of both types of IS is projected to continue rising over the next 3 decades, with diabetes-related IS demonstrating a particularly pronounced upward trend. This projected increase in AF-related IS may be mitigated by advancements in anticoagulant therapies, particularly the broader adoption of direct-acting oral anticoagulants. Specifically, while the prevalence of AF is expected to remain stable globally, the ongoing optimization of anticoagulation strategies is likely to drive a gradual decline in AF-related IS incidence. This finding suggests that the primary shortcoming in current IS management approaches has transitioned from treatment to prevention. Given the previously highlighted role of multimorbidity and interconnected behavioral risk factors, future prevention strategies urgently require a dual approach: first, establishing integrated intervention programmes based on shared disease mechanisms; second, developing a preventive healthcare model connecting hospitals, communities, and households.^33^ Digital platforms can enable real-time sharing of clinical and public health monitoring data, facilitating systematic and coordinated interventions targeting multiple chronic diseases and behavioral risk factors.

This study has several limitations. First, the RRs used in this analysis did not account for heterogeneity across clinical subgroups. For AF, the definition did not differentiate between newly diagnosed and known AF, and for diabetes, it was based on self-report or HbA1c ≥ 6.5% without considering disease duration, glycemic control, or complications. Consequently, our burden estimates capture the overall impact of these risk factors but may not accurately reflect the contributions of specific patient subgroups. Second, because direct RR estimates were unavailable, PAFs were estimated using ORs approximated as RRs, introducing uncertainty in the PAF estimates and, consequently, in the disease burden assessment, which we assessed using Monte Carlo simulations. Third, the analysis assumes a constant RR for diabetes-related IS throughout the study period. In practice, this risk may vary due to improvements in diabetes care or evolving population characteristics, and as a result, our projections may slightly overestimate the future burden of diabetes-related IS. The overall upward trend, however, remains consistent with current evidence. Fourth, the RR for diabetes-related IS was derived from a study that did not adjust for BMI, a known confounder for both diabetes and stroke, which may introduce residual confounding in the observed associations. Finally, estimates from GBD 2021 used in our study may be subject to some uncertainty due to variations in data quality and modeling assumptions, particularly in data-sparse settings. For example, the prevalence of AF in Tanzania from the GBD has 95% UIs, which can influence the estimates of the PAF of IS burden related to AF and the associated disease burden (both current and projected) in Tanzania. These intervals are available via the Global Health Data Exchange online database (http://ghdx.healthdata.org/gbd-results-tool). To account for this uncertainty, we conducted a probabilistic sensitivity analysis using Monte Carlo simulation, sampling prevalence and other key inputs from their 95% UIs. While uncertainty increases for future projections, our findings remained relatively robust. However, this analysis addressed only parameter uncertainty around the selected pooled estimate. Due to data limitations, we could not model potential geographic or temporal heterogeneity in the underlying associations. Consequently, the sensitivity analysis did not fully capture the uncertainty related to the transportability of these estimates across diverse populations and time periods. We, therefore, recommend interpreting absolute estimates with caution and focusing on overall trends.

In conclusion, the burden of AF-related IS remains greater but is declining, whereas the burden attributable to diabetes, although lower at baseline, continues to rise. Given the multifactorial etiology of IS and the frequent co-occurrence of metabolic and behavioral risk factors, these findings support a shift towards integrated prevention strategies that address multimorbidity. In particular, efforts should focus on optimizing AF management in older adults while reducing modifiable behavioral risks, including unhealthy diet and physical inactivity.

## Article Information

### Disclosures

Dr Sivakumar reports receiving compensation as a consultant from Stryker Corporation. The other authors report no conflicts.

### Supplemental Material

Tables S1–S9

Figures S1–S5

GATHER Checklist

## Supplementary Material


