# Supplementary material for: Global Trends in the Burden of Ischemic Stroke Attributable to Diabetes Versus Atrial Fibrillation
Source: Stroke. 2026 Jul 17;57(9):2754–67. doi: 10.1161/STROKEAHA.125.054141 (PMC13506186; doi:10.1161/STROKEAHA.125.054141)
Supplement: Supplementary file 1 [file str-57-2754-s001.pdf]

Supplemental Material

Contents

Table S1. Population attributable fraction of atrial fibrillation on ischaemic stroke in 1990 and 2021 among population aged ≥55 years.

Table S2. Population attributable fraction of diabetes on ischaemic stroke in 1990 and 2021 among population aged ≥55 years.

Table S3. Global and regional burden of atrial fibrillation- versus diabetes-related ischaemic stroke: incidence, mortality, and disability-adjusted life years (1990-2021).

Table S4. Differences in global incidence of ischaemic stroke attributable to atrial fibrillation across age and sex in 2021.

Table S5. Differences in global mortality of ischaemic stroke attributable to atrial fibrillation across age and sex in 2021.

Table S6. Differences in global disability-adjusted life years of ischaemic stroke attributable to atrial fibrillation across age and sex in 2021.

Table S7. Differences in global incidence of ischaemic stroke attributable to diabetes across age and sex in 2021.

Table S8. Differences in global mortality of ischaemic stroke attributable to diabetes across age and sex in 2021.

Table S9. Differences in global disability-adjusted life years of ischaemic stroke attributable to diabetes across age and sex in 2021.

Figure S1. Number of deaths and age-standardized mortality rates for ischaemic

stroke attributable to atrial fibrillation (A) and diabetes (B) by sex in 2021.

Figure S2. Number of disability-adjusted life years and age-standardized disability-adjusted life year rates for ischaemic stroke attributable to atrial fibrillation (A) and diabetes (B) by sex in 2021.

Figure S3. Proportion attributable fraction of atrial fibrillation due to high body mass index, smoking, high alcohol consumption, and dietary risks across regions in 2021.

Figure S4. Proportion attributable fraction of diabetes due to high body mass index, smoking, high alcohol consumption, and dietary risks across regions in 2021.

Figure S5. Proportion attributable fraction of ischaemic stroke due to high body mass index, smoking, high alcohol consumption, and dietary risks across regions in 2021.

Table S1. Population attributable fraction of atrial fibrillation on ischaemic stroke in 1990 and 2021 among population aged ≥55 years.

**Location**	**1990 PAF**	**Lower**	**Upper**	**2021 PAF**	**Lower**	**Upper**
Global	0.10	0.09	0.12	0.10	0.10	0.13
Region						
Advanced Health System	0.13	0.12	0.15	0.14	0.13	0.17
Africa	0.06	0.05	0.07	0.06	0.06	0.08
African Region	0.06	0.06	0.08	0.06	0.06	0.08
America	0.14	0.13	0.17	0.14	0.13	0.18
Andean Latin America	0.10	0.09	0.12	0.11	0.10	0.13
Asia	0.07	0.07	0.09	0.09	0.08	0.10
Australasia	0.14	0.13	0.18	0.16	0.15	0.20
Basic Health System	0.07	0.07	0.09	0.09	0.08	0.11
Caribbean	0.11	0.10	0.14	0.11	0.11	0.14
Central Africa	0.06	0.05	0.07	0.06	0.05	0.07
Central Asia	0.08	0.08	0.10	0.08	0.07	0.10
Central Europe	0.10	0.09	0.12	0.13	0.12	0.15
Central Latin America	0.11	0.10	0.13	0.11	0.11	0.14
Central Sub-Saharan Africa	0.06	0.05	0.07	0.06	0.05	0.07
Commonwealth High Income	0.14	0.13	0.17	0.14	0.13	0.17
Commonwealth Low Income	0.07	0.06	0.08	0.07	0.07	0.09

Commonwealth Middle Income	0.07	0.07	0.09	0.08	0.08	0.10
East Asia	0.06	0.06	0.08	0.08	0.08	0.10
East Asia & Pacific - WB	0.07	0.07	0.09	0.09	0.08	0.11
Eastern Africa	0.06	0.05	0.07	0.07	0.06	0.08
Eastern Europe	0.09	0.09	0.12	0.11	0.10	0.13
Eastern Mediterranean Region	0.06	0.06	0.08	0.06	0.06	0.08
Eastern Sub-Saharan Africa	0.06	0.05	0.07	0.07	0.06	0.08
Europe	0.12	0.11	0.15	0.14	0.13	0.17
Europe & Central Asia - WB	0.12	0.11	0.15	0.13	0.13	0.16
European Region	0.12	0.11	0.15	0.13	0.13	0.16
High-income Asia Pacific	0.08	0.08	0.10	0.09	0.08	0.11
High-income North America	0.16	0.15	0.19	0.18	0.16	0.22
Latin America & Caribbean - WB	0.11	0.10	0.13	0.11	0.10	0.14
Limited Health System	0.07	0.07	0.09	0.08	0.07	0.10
Middle East & North Africa - WB	0.05	0.05	0.07	0.06	0.06	0.08
Minimal Health System	0.06	0.05	0.07	0.06	0.05	0.07
North Africa and Middle East	0.05	0.05	0.06	0.05	0.05	0.07
North America	0.16	0.15	0.19	0.18	0.16	0.22
Northern Africa	0.05	0.05	0.06	0.05	0.05	0.07
Oceania	0.07	0.07	0.09	0.08	0.07	0.09
Region of the Americas	0.14	0.13	0.17	0.14	0.13	0.18

South Asia	0.07	0.07	0.09	0.08	0.08	0.10
South Asia - WB	0.07	0.07	0.09	0.08	0.08	0.10
Southeast Asia	0.09	0.08	0.11	0.10	0.09	0.12
South-East Asia Region	0.08	0.07	0.09	0.09	0.08	0.11
Southern Africa	0.07	0.07	0.09	0.07	0.07	0.09
Southern Latin America	0.08	0.07	0.10	0.07	0.07	0.09
Southern Sub-Saharan Africa	0.08	0.07	0.10	0.08	0.07	0.09
Sub-Saharan Africa - WB	0.06	0.06	0.08	0.07	0.06	0.08
Tropical Latin America	0.12	0.11	0.14	0.12	0.12	0.15
Western Africa	0.06	0.06	0.07	0.06	0.06	0.08
Western Europe	0.14	0.13	0.18	0.16	0.15	0.20
Western Pacific Region	0.07	0.07	0.09	0.09	0.08	0.10
Western Sub-Saharan Africa	0.06	0.06	0.07	0.06	0.06	0.08
SDI						
High SDI	0.13	0.12	0.16	0.14	0.13	0.18
High-middle SDI	0.09	0.08	0.11	0.10	0.09	0.12
Middle SDI	0.08	0.07	0.09	0.09	0.08	0.11
Low-middle SDI	0.08	0.07	0.09	0.08	0.08	0.10
Low SDI	0.06	0.06	0.08	0.07	0.06	0.08
Country						
Afghanistan	0.04	0.04	0.05	0.06	0.05	0.07

Albania	0.09	0.08	0.11	0.10	0.09	0.12
Algeria	0.05	0.05	0.06	0.06	0.05	0.07
American Samoa	0.08	0.08	0.10	0.10	0.09	0.12
Andorra	0.13	0.12	0.16	0.13	0.12	0.16
Angola	0.06	0.05	0.07	0.06	0.06	0.07
Antigua and Barbuda	0.12	0.11	0.15	0.10	0.09	0.12
Argentina	0.08	0.07	0.10	0.06	0.06	0.08
Armenia	0.08	0.07	0.09	0.09	0.09	0.12
Australia	0.15	0.14	0.18	0.16	0.15	0.20
Austria	0.12	0.11	0.15	0.22	0.20	0.27
Azerbaijan	0.08	0.07	0.09	0.07	0.07	0.09
Bahrain	0.04	0.04	0.05	0.04	0.04	0.05
Bangladesh	0.08	0.07	0.09	0.08	0.08	0.10
Barbados	0.13	0.12	0.16	0.12	0.11	0.15
Belarus	0.09	0.08	0.11	0.10	0.10	0.13
Belgium	0.13	0.12	0.15	0.12	0.12	0.15
Belize	0.11	0.11	0.14	0.10	0.10	0.13
Benin	0.06	0.06	0.08	0.06	0.06	0.07
Bermuda	0.11	0.11	0.14	0.13	0.12	0.16
Bhutan	0.06	0.06	0.08	0.08	0.08	0.10
Bolivarian Republic of Venezuela	0.12	0.11	0.14	0.11	0.10	0.14

Bosnia and Herzegovina	0.08	0.07	0.10	0.10	0.10	0.12
Botswana	0.06	0.06	0.08	0.07	0.06	0.08
Brazil	0.12	0.11	0.14	0.13	0.12	0.15
Brunei Darussalam	0.08	0.07	0.10	0.06	0.06	0.08
Bulgaria	0.09	0.09	0.12	0.11	0.10	0.13
Burkina Faso	0.06	0.05	0.07	0.06	0.06	0.07
Burundi	0.06	0.06	0.08	0.06	0.05	0.07
Cambodia	0.08	0.07	0.10	0.08	0.07	0.10
Cameroon	0.06	0.05	0.07	0.06	0.06	0.08
Canada	0.19	0.18	0.23	0.17	0.16	0.21
Central African Republic	0.05	0.05	0.07	0.05	0.05	0.06
Chad	0.06	0.06	0.07	0.06	0.05	0.07
Chile	0.09	0.08	0.10	0.09	0.08	0.11
China	0.06	0.06	0.08	0.08	0.08	0.10
Colombia	0.10	0.09	0.12	0.11	0.10	0.13
Commonwealth of the Bahamas	0.11	0.10	0.14	0.10	0.09	0.12
Comoros	0.06	0.05	0.07	0.06	0.06	0.08
Congo	0.06	0.06	0.07	0.06	0.06	0.08
Cook Islands	0.09	0.08	0.11	0.11	0.10	0.14
Costa Rica	0.12	0.11	0.15	0.12	0.11	0.15
Croatia	0.06	0.06	0.07	0.08	0.08	0.10

Cuba	0.12	0.11	0.14	0.12	0.11	0.14
Cyprus	0.11	0.10	0.13	0.09	0.08	0.11
Czech Republic	0.12	0.11	0.14	0.18	0.17	0.22
Democratic People's Republic of Korea	0.07	0.07	0.09	0.08	0.08	0.10
Democratic Republic of the Congo	0.06	0.05	0.07	0.06	0.05	0.07
Denmark	0.15	0.14	0.18	0.16	0.15	0.20
Djibouti	0.05	0.05	0.06	0.06	0.05	0.07
Dominica	0.12	0.11	0.14	0.11	0.10	0.13
Dominican Republic	0.10	0.10	0.13	0.11	0.10	0.14
Ecuador	0.10	0.09	0.12	0.10	0.10	0.13
Egypt	0.04	0.04	0.05	0.05	0.05	0.06
El Salvador	0.11	0.10	0.13	0.12	0.11	0.15
Equatorial Guinea	0.06	0.05	0.07	0.07	0.06	0.09
Eritrea	0.04	0.04	0.06	0.05	0.05	0.06
Estonia	0.09	0.09	0.11	0.12	0.11	0.14
Ethiopia	0.06	0.05	0.07	0.07	0.07	0.09
Federated States of Micronesia	0.09	0.08	0.11	0.07	0.07	0.09
Fiji	0.08	0.08	0.10	0.09	0.08	0.11
Finland	0.19	0.18	0.23	0.17	0.16	0.20
France	0.14	0.14	0.18	0.14	0.13	0.17

Gabon	0.07	0.06	0.08	0.07	0.06	0.08
Georgia	0.09	0.08	0.11	0.10	0.10	0.12
Germany	0.16	0.15	0.20	0.19	0.17	0.23
Ghana	0.06	0.05	0.07	0.06	0.06	0.08
Greece	0.12	0.11	0.14	0.14	0.13	0.17
Greenland	0.14	0.13	0.17	0.13	0.12	0.16
Grenada	0.12	0.11	0.15	0.10	0.09	0.12
Guam	0.08	0.07	0.10	0.11	0.10	0.14
Guatemala	0.09	0.08	0.11	0.10	0.10	0.13
Guinea	0.06	0.06	0.07	0.06	0.06	0.07
Guinea-Bissau	0.06	0.05	0.07	0.05	0.05	0.07
Guyana	0.10	0.10	0.13	0.10	0.09	0.12
Haiti	0.09	0.08	0.11	0.09	0.08	0.11
Honduras	0.10	0.09	0.12	0.10	0.09	0.12
Hungary	0.12	0.11	0.14	0.11	0.10	0.14
Iceland	0.13	0.12	0.16	0.14	0.13	0.17
India	0.07	0.07	0.09	0.08	0.08	0.10
Indonesia	0.10	0.09	0.12	0.10	0.09	0.12
Iraq	0.06	0.06	0.08	0.06	0.05	0.07
Ireland	0.13	0.12	0.16	0.12	0.11	0.15
Islamic Republic of Iran	0.05	0.05	0.06	0.07	0.06	0.08

Israel	0.15	0.14	0.18	0.20	0.18	0.24
Italy	0.15	0.14	0.19	0.17	0.16	0.20
Jamaica	0.12	0.11	0.14	0.12	0.11	0.14
Japan	0.08	0.08	0.10	0.09	0.08	0.11
Jordan	0.05	0.05	0.06	0.05	0.05	0.07
Kazakhstan	0.09	0.08	0.11	0.09	0.08	0.11
Kenya	0.07	0.06	0.09	0.07	0.06	0.08
Kingdom of Eswatini	0.07	0.06	0.08	0.07	0.06	0.08
Kiribati	0.08	0.07	0.10	0.08	0.07	0.09
Kuwait	0.05	0.05	0.06	0.06	0.05	0.07
Kyrgyzstan	0.08	0.07	0.09	0.07	0.07	0.09
Lao People's Democratic Republic	0.08	0.08	0.10	0.09	0.08	0.10
Latvia	0.09	0.08	0.11	0.12	0.11	0.14
Lebanon	0.05	0.05	0.06	0.08	0.07	0.09
Lesotho	0.06	0.06	0.08	0.06	0.06	0.08
Liberia	0.06	0.06	0.08	0.06	0.06	0.07
Libya	0.06	0.05	0.07	0.06	0.06	0.07
Lithuania	0.09	0.09	0.12	0.12	0.11	0.14
Luxembourg	0.13	0.12	0.16	0.14	0.13	0.17
Madagascar	0.06	0.05	0.07	0.05	0.05	0.07
Malawi	0.06	0.05	0.07	0.07	0.06	0.08

Malaysia	0.10	0.09	0.12	0.10	0.09	0.12
Maldives	0.08	0.07	0.09	0.09	0.08	0.11
Mali	0.05	0.05	0.06	0.06	0.05	0.07
Malta	0.11	0.11	0.14	0.12	0.11	0.15
Marshall Islands	0.08	0.07	0.10	0.07	0.07	0.09
Mauritania	0.06	0.06	0.07	0.07	0.06	0.08
Mauritius	0.09	0.08	0.11	0.10	0.09	0.12
Mexico	0.11	0.10	0.14	0.12	0.11	0.14
Mongolia	0.08	0.08	0.10	0.08	0.07	0.09
Montenegro	0.10	0.09	0.12	0.10	0.10	0.12
Morocco	0.06	0.05	0.07	0.06	0.06	0.07
Mozambique	0.06	0.05	0.07	0.06	0.06	0.07
Myanmar	0.09	0.08	0.10	0.09	0.08	0.11
Namibia	0.06	0.06	0.08	0.07	0.06	0.08
Nepal	0.07	0.06	0.08	0.07	0.07	0.09
Netherlands	0.15	0.14	0.18	0.14	0.13	0.18
New Zealand	0.14	0.13	0.17	0.14	0.14	0.18
Nicaragua	0.10	0.09	0.12	0.10	0.10	0.13
Niger	0.05	0.05	0.06	0.05	0.05	0.07
Nigeria	0.06	0.06	0.08	0.07	0.06	0.08
Northern Mariana Islands	0.08	0.07	0.10	0.08	0.08	0.10

Norway	0.15	0.14	0.19	0.14	0.13	0.17
Oman	0.04	0.04	0.05	0.05	0.04	0.06
Pakistan	0.09	0.08	0.11	0.08	0.07	0.10
Palestine	0.05	0.05	0.06	0.05	0.05	0.06
Panama	0.11	0.10	0.14	0.12	0.11	0.15
Papua New Guinea	0.07	0.06	0.08	0.07	0.07	0.09
Paraguay	0.12	0.11	0.15	0.12	0.11	0.14
Peru	0.10	0.09	0.12	0.11	0.11	0.14
Philippines	0.09	0.09	0.11	0.09	0.09	0.12
Plurinational State of Bolivia	0.09	0.08	0.11	0.10	0.09	0.12
Poland	0.11	0.10	0.13	0.15	0.14	0.19
Portugal	0.13	0.12	0.16	0.15	0.14	0.18
Principality of Monaco	0.16	0.15	0.19	0.14	0.13	0.17
Puerto Rico	0.12	0.11	0.15	0.14	0.13	0.17
Qatar	0.04	0.04	0.05	0.04	0.04	0.05
Republic of Cabo Verde	0.07	0.07	0.09	0.07	0.06	0.08
Republic of Côte d'Ivoire	0.05	0.05	0.07	0.06	0.06	0.07
Republic of Korea	0.08	0.08	0.10	0.10	0.09	0.12
Republic of Moldova	0.09	0.08	0.11	0.10	0.10	0.13
Republic of Nauru	0.08	0.08	0.10	0.09	0.08	0.11
Republic of Niue	0.12	0.11	0.14	0.11	0.10	0.13

Republic of Palau	0.09	0.08	0.11	0.08	0.08	0.10
Republic of San Marino	0.14	0.13	0.17	0.14	0.13	0.18
Republic of the Gambia	0.06	0.06	0.07	0.06	0.06	0.08
Romania	0.09	0.09	0.11	0.08	0.08	0.10
Russian Federation	0.10	0.09	0.12	0.11	0.11	0.14
Rwanda	0.06	0.05	0.07	0.06	0.06	0.08
Saint Kitts and Nevis	0.12	0.11	0.14	0.09	0.08	0.11
Saint Lucia	0.12	0.11	0.14	0.11	0.10	0.14
Saint Vincent and the Grenadines	0.11	0.10	0.13	0.11	0.10	0.13
Samoa	0.09	0.08	0.11	0.09	0.09	0.12
Sao Tome and Principe	0.06	0.06	0.08	0.06	0.06	0.08
Saudi Arabia	0.05	0.05	0.06	0.05	0.04	0.06
Senegal	0.06	0.06	0.08	0.06	0.06	0.08
Serbia	0.08	0.08	0.10	0.08	0.07	0.10
Seychelles	0.11	0.10	0.13	0.10	0.09	0.12
Sierra Leone	0.07	0.07	0.09	0.07	0.06	0.08
Singapore	0.07	0.07	0.09	0.07	0.06	0.08
Slovakia	0.14	0.13	0.17	0.16	0.15	0.19
Slovenia	0.10	0.10	0.13	0.12	0.11	0.14
Socialist Republic of Viet Nam	0.09	0.08	0.11	0.09	0.09	0.12
Solomon Islands	0.07	0.07	0.09	0.08	0.07	0.10

Somalia	0.05	0.05	0.06	0.05	0.05	0.06
South Africa	0.09	0.08	0.10	0.08	0.08	0.10
South Sudan	0.06	0.06	0.07	0.06	0.05	0.07
Spain	0.15	0.14	0.18	0.16	0.15	0.20
Sri Lanka	0.09	0.08	0.11	0.10	0.09	0.12
Sudan	0.05	0.05	0.06	0.06	0.06	0.07
Suriname	0.10	0.09	0.12	0.10	0.10	0.12
Sweden	0.19	0.18	0.23	0.27	0.25	0.33
Switzerland	0.09	0.09	0.11	0.09	0.08	0.11
Syrian Arab Republic	0.05	0.05	0.06	0.05	0.05	0.06
Taiwan (Province of China)	0.09	0.09	0.11	0.11	0.10	0.13
Tajikistan	0.08	0.07	0.09	0.07	0.06	0.08
Thailand	0.08	0.08	0.10	0.10	0.09	0.12
The former Yugoslav Republic of Macedonia	0.09	0.09	0.11	0.09	0.09	0.12
Timor-Leste	0.08	0.07	0.09	0.09	0.09	0.11
Togo	0.06	0.06	0.07	0.06	0.05	0.07
Tokelau	0.09	0.08	0.11	0.11	0.10	0.13
Tonga	0.09	0.08	0.11	0.11	0.10	0.13
Trinidad and Tobago	0.12	0.11	0.14	0.12	0.11	0.14
Tunisia	0.05	0.05	0.06	0.06	0.06	0.07

Turkey	0.05	0.05	0.06	0.05	0.04	0.06
Turkmenistan	0.08	0.07	0.09	0.08	0.08	0.10
Tuvalu	0.07	0.07	0.09	0.09	0.08	0.11
Uganda	0.07	0.06	0.08	0.06	0.06	0.08
Ukraine	0.09	0.09	0.11	0.10	0.10	0.13
United Arab Emirates	0.05	0.05	0.06	0.03	0.03	0.04
United Kingdom of Great Britain and Northern Ireland	0.12	0.11	0.14	0.12	0.12	0.15
United Republic of Tanzania	0.06	0.05	0.07	0.07	0.06	0.08
United States of America	0.16	0.15	0.19	0.18	0.17	0.22
United States Virgin Islands	0.10	0.10	0.13	0.13	0.12	0.15
Uruguay	0.09	0.08	0.10	0.09	0.08	0.11
Uzbekistan	0.08	0.07	0.09	0.07	0.07	0.09
Vanuatu	0.09	0.08	0.11	0.09	0.08	0.11
Yemen	0.04	0.04	0.05	0.05	0.05	0.06
Zambia	0.06	0.05	0.07	0.06	0.05	0.07
Zimbabwe	0.06	0.06	0.08	0.06	0.06	0.08

PAF = population attributable fraction.

Table S2. Population attributable fraction of diabetes on ischaemic stroke in 1990 and 2021 among population aged ≥55 years.

**Location**	**1990 PAF**	**Lower**	**Upper**	**2021 PAF**	**Lower**	**Upper**
Global	0.03	0.03	0.04	0.06	0.06	0.08
Region						
Advanced Health System	0.03	0.03	0.04	0.07	0.07	0.09
Africa	0.03	0.03	0.03	0.06	0.06	0.08
African Region	0.03	0.02	0.03	0.05	0.05	0.06
America	0.05	0.04	0.06	0.09	0.08	0.10
Andean Latin America	0.03	0.03	0.03	0.06	0.06	0.07
Asia	0.03	0.03	0.04	0.06	0.05	0.07
Australasia	0.03	0.02	0.03	0.05	0.04	0.06
Basic Health System	0.04	0.03	0.05	0.06	0.06	0.07
Caribbean	0.05	0.05	0.07	0.09	0.08	0.11
Central Africa	0.03	0.02	0.03	0.05	0.04	0.06
Central Asia	0.02	0.02	0.03	0.05	0.05	0.07
Central Europe	0.04	0.04	0.05	0.07	0.06	0.08
Central Latin America	0.06	0.06	0.07	0.08	0.07	0.10
Central Sub-Saharan Africa	0.03	0.02	0.03	0.05	0.05	0.06
Commonwealth High Income	0.03	0.03	0.04	0.07	0.06	0.08
Commonwealth Low Income	0.02	0.02	0.03	0.05	0.05	0.06

Commonwealth Middle Income	0.03	0.03	0.04	0.06	0.05	0.07
East Asia	0.04	0.03	0.04	0.05	0.05	0.06
East Asia & Pacific - WB	0.04	0.03	0.04	0.06	0.05	0.07
Eastern Africa	0.02	0.02	0.03	0.04	0.04	0.05
Eastern Europe	0.02	0.02	0.03	0.04	0.04	0.05
Eastern Mediterranean Region	0.04	0.04	0.05	0.09	0.09	0.11
Eastern Sub-Saharan Africa	0.02	0.02	0.02	0.03	0.03	0.04
Europe	0.03	0.03	0.04	0.05	0.05	0.07
Europe & Central Asia - WB	0.03	0.03	0.04	0.05	0.05	0.07
European Region	0.03	0.03	0.04	0.05	0.05	0.07
High-income Asia Pacific	0.04	0.04	0.05	0.08	0.07	0.09
High-income North America	0.04	0.04	0.05	0.10	0.09	0.12
Latin America & Caribbean - WB	0.05	0.05	0.06	0.07	0.07	0.09
Limited Health System	0.03	0.03	0.04	0.05	0.05	0.07
Middle East & North Africa - WB	0.04	0.04	0.05	0.10	0.09	0.12
Minimal Health System	0.03	0.03	0.03	0.05	0.05	0.07
North Africa and Middle East	0.04	0.04	0.05	0.10	0.09	0.12
North America	0.04	0.04	0.05	0.10	0.09	0.12
Northern Africa	0.04	0.04	0.05	0.11	0.10	0.13
Oceania	0.05	0.05	0.06	0.11	0.10	0.13
Region of the Americas	0.05	0.04	0.06	0.09	0.08	0.10

South Asia	0.03	0.03	0.04	0.06	0.05	0.07
South Asia - WB	0.03	0.03	0.04	0.06	0.05	0.07
Southeast Asia	0.03	0.03	0.04	0.06	0.06	0.08
South-East Asia Region	0.03	0.03	0.04	0.06	0.05	0.07
Southern Africa	0.03	0.03	0.03	0.06	0.05	0.07
Southern Latin America	0.04	0.03	0.04	0.07	0.07	0.09
Southern Sub-Saharan Africa	0.03	0.03	0.04	0.07	0.06	0.08
Sub-Saharan Africa - WB	0.02	0.02	0.03	0.05	0.04	0.06
Tropical Latin America	0.05	0.04	0.06	0.06	0.06	0.08
Western Africa	0.03	0.02	0.03	0.05	0.05	0.06
Western Europe	0.03	0.03	0.04	0.06	0.05	0.07
Western Pacific Region	0.04	0.03	0.04	0.05	0.05	0.07
Western Sub-Saharan Africa	0.03	0.02	0.03	0.05	0.05	0.06
SDI						
High SDI	0.04	0.03	0.05	0.08	0.07	0.09
High-middle SDI	0.03	0.03	0.04	0.06	0.05	0.07
Middle SDI	0.04	0.03	0.04	0.06	0.05	0.07
Low-middle SDI	0.03	0.03	0.04	0.06	0.06	0.07
Low SDI	0.03	0.02	0.03	0.05	0.05	0.06
Country						
Afghanistan	0.06	0.05	0.07	0.14	0.13	0.17

Albania	0.03	0.03	0.04	0.04	0.04	0.05
Algeria	0.05	0.04	0.06	0.11	0.10	0.13
American Samoa	0.08	0.08	0.10	0.16	0.15	0.20
Andorra	0.02	0.02	0.03	0.05	0.05	0.06
Angola	0.03	0.03	0.04	0.06	0.05	0.07
Antigua and Barbuda	0.07	0.07	0.09	0.11	0.10	0.14
Argentina	0.04	0.03	0.05	0.07	0.06	0.08
Armenia	0.03	0.03	0.04	0.05	0.05	0.06
Australia	0.03	0.02	0.03	0.05	0.04	0.06
Austria	0.02	0.02	0.02	0.03	0.03	0.04
Azerbaijan	0.02	0.02	0.03	0.05	0.05	0.06
Bahrain	0.08	0.08	0.10	0.16	0.15	0.19
Bangladesh	0.03	0.03	0.04	0.06	0.06	0.08
Barbados	0.07	0.07	0.09	0.10	0.10	0.13
Belarus	0.02	0.02	0.02	0.03	0.03	0.04
Belgium	0.03	0.02	0.03	0.05	0.05	0.06
Belize	0.05	0.05	0.06	0.09	0.08	0.11
Benin	0.03	0.03	0.04	0.06	0.06	0.08
Bermuda	0.04	0.04	0.05	0.07	0.06	0.08
Bhutan	0.03	0.02	0.03	0.05	0.05	0.06
Bolivarian Republic of Venezuela	0.04	0.04	0.05	0.07	0.07	0.09

Bosnia and Herzegovina	0.04	0.04	0.05	0.09	0.08	0.11
Botswana	0.03	0.02	0.03	0.06	0.05	0.07
Brazil	0.05	0.04	0.06	0.06	0.06	0.08
Brunei Darussalam	0.07	0.07	0.09	0.14	0.13	0.17
Bulgaria	0.04	0.04	0.05	0.07	0.07	0.09
Burkina Faso	0.02	0.02	0.03	0.05	0.04	0.06
Burundi	0.02	0.02	0.02	0.03	0.03	0.04
Cambodia	0.02	0.02	0.03	0.06	0.05	0.07
Cameroon	0.02	0.02	0.03	0.05	0.05	0.06
Canada	0.03	0.03	0.04	0.08	0.08	0.10
Central African Republic	0.03	0.03	0.04	0.06	0.06	0.08
Chad	0.03	0.02	0.03	0.05	0.05	0.06
Chile	0.04	0.03	0.05	0.08	0.08	0.10
China	0.04	0.03	0.04	0.05	0.05	0.06
Colombia	0.04	0.04	0.05	0.06	0.06	0.08
Commonwealth of the Bahamas	0.06	0.05	0.07	0.10	0.09	0.12
Comoros	0.02	0.02	0.03	0.05	0.04	0.06
Congo	0.03	0.03	0.03	0.06	0.05	0.07
Cook Islands	0.09	0.08	0.11	0.15	0.14	0.19
Costa Rica	0.05	0.04	0.06	0.08	0.07	0.09
Croatia	0.04	0.04	0.05	0.07	0.06	0.08

Cuba	0.04	0.04	0.05	0.06	0.06	0.08
Cyprus	0.07	0.06	0.08	0.07	0.07	0.09
Czech Republic	0.04	0.04	0.05	0.07	0.07	0.09
Democratic People's Republic of Korea	0.03	0.02	0.03	0.05	0.05	0.06
Democratic Republic of the Congo	0.02	0.02	0.03	0.05	0.04	0.06
Denmark	0.02	0.02	0.02	0.04	0.04	0.05
Djibouti	0.01	0.01	0.02	0.03	0.03	0.04
Dominica	0.07	0.07	0.09	0.11	0.10	0.14
Dominican Republic	0.04	0.04	0.05	0.09	0.08	0.11
Ecuador	0.04	0.03	0.05	0.08	0.08	0.10
Egypt	0.02	0.02	0.03	0.09	0.08	0.11
El Salvador	0.03	0.03	0.04	0.07	0.06	0.08
Equatorial Guinea	0.03	0.03	0.03	0.06	0.06	0.08
Eritrea	0.02	0.02	0.02	0.04	0.04	0.05
Estonia	0.02	0.02	0.03	0.05	0.04	0.06
Ethiopia	0.02	0.02	0.03	0.04	0.03	0.04
Federated States of Micronesia	0.06	0.05	0.07	0.12	0.11	0.15
Fiji	0.07	0.07	0.09	0.15	0.14	0.18
Finland	0.04	0.03	0.04	0.07	0.06	0.08
France	0.02	0.02	0.03	0.04	0.04	0.05

Gabon	0.03	0.03	0.04	0.07	0.06	0.08
Georgia	0.02	0.02	0.03	0.06	0.05	0.07
Germany	0.02	0.02	0.03	0.05	0.05	0.06
Ghana	0.03	0.02	0.03	0.05	0.05	0.07
Greece	0.03	0.03	0.04	0.05	0.05	0.06
Greenland	0.01	0.01	0.01	0.04	0.04	0.05
Grenada	0.07	0.07	0.09	0.12	0.11	0.14
Guam	0.04	0.04	0.05	0.07	0.07	0.09
Guatemala	0.04	0.03	0.04	0.09	0.08	0.11
Guinea	0.02	0.02	0.03	0.05	0.04	0.06
Guinea-Bissau	0.03	0.03	0.04	0.06	0.05	0.07
Guyana	0.09	0.09	0.11	0.14	0.13	0.17
Haiti	0.07	0.06	0.08	0.11	0.10	0.13
Honduras	0.04	0.04	0.05	0.08	0.08	0.10
Hungary	0.04	0.04	0.05	0.07	0.06	0.08
Iceland	0.02	0.02	0.03	0.05	0.04	0.06
India	0.03	0.03	0.04	0.05	0.05	0.06
Indonesia	0.03	0.02	0.03	0.05	0.05	0.06
Iraq	0.07	0.07	0.09	0.14	0.13	0.18
Ireland	0.03	0.02	0.03	0.04	0.04	0.05
Islamic Republic of Iran	0.03	0.03	0.04	0.08	0.07	0.10

Israel	0.04	0.04	0.05	0.06	0.06	0.08
Italy	0.04	0.04	0.05	0.05	0.05	0.06
Jamaica	0.06	0.05	0.07	0.09	0.08	0.11
Japan	0.04	0.04	0.05	0.07	0.06	0.08
Jordan	0.08	0.07	0.10	0.14	0.13	0.17
Kazakhstan	0.03	0.03	0.04	0.06	0.06	0.08
Kenya	0.01	0.01	0.02	0.02	0.02	0.03
Kingdom of Eswatini	0.03	0.03	0.04	0.08	0.07	0.10
Kiribati	0.06	0.06	0.08	0.12	0.11	0.15
Kuwait	0.08	0.07	0.10	0.15	0.14	0.18
Kyrgyzstan	0.02	0.02	0.02	0.04	0.04	0.05
Lao People's Democratic Republic	0.03	0.03	0.04	0.07	0.06	0.08
Latvia	0.02	0.02	0.03	0.05	0.04	0.06
Lebanon	0.06	0.05	0.07	0.12	0.11	0.14
Lesotho	0.02	0.02	0.03	0.06	0.06	0.07
Liberia	0.03	0.03	0.04	0.06	0.05	0.07
Libya	0.04	0.04	0.05	0.11	0.10	0.13
Lithuania	0.02	0.02	0.02	0.04	0.04	0.05
Luxembourg	0.03	0.02	0.03	0.05	0.04	0.06
Madagascar	0.02	0.01	0.02	0.03	0.03	0.03
Malawi	0.01	0.01	0.02	0.02	0.02	0.03

Malaysia	0.05	0.05	0.06	0.09	0.08	0.11
Maldives	0.03	0.03	0.04	0.06	0.06	0.08
Mali	0.04	0.04	0.05	0.09	0.08	0.11
Malta	0.03	0.03	0.04	0.07	0.07	0.09
Marshall Islands	0.09	0.08	0.11	0.16	0.15	0.20
Mauritania	0.02	0.02	0.03	0.04	0.03	0.05
Mauritius	0.06	0.06	0.08	0.12	0.12	0.15
Mexico	0.08	0.07	0.10	0.09	0.08	0.11
Mongolia	0.02	0.02	0.02	0.04	0.03	0.04
Montenegro	0.05	0.04	0.06	0.07	0.07	0.09
Morocco	0.05	0.05	0.07	0.14	0.13	0.17
Mozambique	0.02	0.01	0.02	0.03	0.03	0.04
Myanmar	0.04	0.04	0.05	0.09	0.08	0.11
Namibia	0.03	0.02	0.03	0.05	0.05	0.06
Nepal	0.03	0.03	0.04	0.06	0.06	0.08
Netherlands	0.03	0.03	0.03	0.04	0.04	0.05
New Zealand	0.03	0.03	0.04	0.05	0.05	0.06
Nicaragua	0.05	0.05	0.06	0.09	0.08	0.11
Niger	0.03	0.02	0.03	0.05	0.05	0.06
Nigeria	0.02	0.02	0.03	0.04	0.04	0.05
Northern Mariana Islands	0.04	0.04	0.05	0.08	0.08	0.10

Norway	0.04	0.03	0.04	0.05	0.04	0.06
Oman	0.05	0.05	0.06	0.09	0.09	0.12
Pakistan	0.03	0.03	0.04	0.07	0.06	0.08
Palestine	0.05	0.05	0.07	0.11	0.10	0.13
Panama	0.04	0.04	0.05	0.08	0.07	0.09
Papua New Guinea	0.05	0.04	0.06	0.10	0.09	0.12
Paraguay	0.04	0.04	0.05	0.08	0.07	0.09
Peru	0.02	0.02	0.03	0.04	0.04	0.05
Philippines	0.04	0.04	0.05	0.05	0.05	0.06
Plurinational State of Bolivia	0.03	0.03	0.04	0.07	0.07	0.09
Poland	0.04	0.04	0.05	0.07	0.06	0.08
Portugal	0.04	0.04	0.05	0.08	0.07	0.10
Principality of Monaco	0.02	0.02	0.02	0.04	0.04	0.05
Puerto Rico	0.07	0.07	0.09	0.12	0.11	0.14
Qatar	0.08	0.07	0.09	0.16	0.15	0.19
Republic of Cabo Verde	0.03	0.03	0.04	0.07	0.07	0.09
Republic of Côte d'Ivoire	0.03	0.03	0.03	0.06	0.05	0.07
Republic of Korea	0.04	0.04	0.05	0.10	0.09	0.12
Republic of Moldova	0.03	0.03	0.03	0.05	0.05	0.06
Republic of Nauru	0.07	0.06	0.08	0.13	0.12	0.16
Republic of Niue	0.08	0.07	0.09	0.14	0.13	0.18

Republic of Palau	0.07	0.07	0.09	0.14	0.13	0.17
Republic of San Marino	0.02	0.02	0.03	0.05	0.04	0.06
Republic of the Gambia	0.03	0.02	0.03	0.05	0.05	0.07
Romania	0.03	0.03	0.03	0.05	0.04	0.06
Russian Federation	0.02	0.02	0.03	0.05	0.04	0.06
Rwanda	0.02	0.01	0.02	0.02	0.02	0.03
Saint Kitts and Nevis	0.07	0.07	0.09	0.10	0.10	0.13
Saint Lucia	0.09	0.09	0.12	0.12	0.11	0.15
Saint Vincent and the Grenadines	0.09	0.08	0.11	0.12	0.11	0.15
Samoa	0.07	0.06	0.08	0.13	0.12	0.15
Sao Tome and Principe	0.03	0.03	0.04	0.06	0.05	0.07
Saudi Arabia	0.06	0.05	0.07	0.11	0.10	0.13
Senegal	0.04	0.04	0.05	0.08	0.07	0.09
Serbia	0.05	0.05	0.06	0.08	0.08	0.10
Seychelles	0.04	0.04	0.05	0.12	0.11	0.15
Sierra Leone	0.03	0.02	0.03	0.05	0.05	0.06
Singapore	0.06	0.06	0.08	0.08	0.07	0.10
Slovakia	0.04	0.03	0.05	0.05	0.05	0.06
Slovenia	0.04	0.04	0.05	0.05	0.05	0.07
Socialist Republic of Viet Nam	0.03	0.03	0.03	0.05	0.05	0.07
Solomon Islands	0.03	0.03	0.04	0.08	0.07	0.09

Somalia	0.02	0.02	0.02	0.03	0.03	0.04
South Africa	0.03	0.03	0.04	0.07	0.07	0.09
South Sudan	0.02	0.02	0.02	0.03	0.03	0.04
Spain	0.05	0.05	0.06	0.08	0.07	0.09
Sri Lanka	0.05	0.05	0.07	0.12	0.11	0.15
Sudan	0.04	0.04	0.05	0.08	0.08	0.10
Suriname	0.06	0.06	0.07	0.12	0.11	0.14
Sweden	0.03	0.03	0.04	0.05	0.05	0.07
Switzerland	0.04	0.04	0.05	0.07	0.07	0.09
Syrian Arab Republic	0.04	0.04	0.05	0.09	0.09	0.11
Taiwan (Province of China)	0.04	0.04	0.05	0.06	0.06	0.08
Tajikistan	0.02	0.02	0.02	0.04	0.04	0.05
Thailand	0.03	0.03	0.04	0.06	0.06	0.08
The former Yugoslav Republic of Macedonia	0.05	0.05	0.06	0.09	0.08	0.11
Timor-Leste	0.02	0.02	0.02	0.07	0.07	0.09
Togo	0.02	0.02	0.02	0.04	0.03	0.05
Tokelau	0.08	0.07	0.10	0.14	0.13	0.17
Tonga	0.07	0.06	0.08	0.12	0.11	0.15
Trinidad and Tobago	0.10	0.09	0.12	0.14	0.13	0.17
Tunisia	0.04	0.04	0.05	0.11	0.10	0.13

Turkey	0.03	0.03	0.04	0.07	0.07	0.09
Turkmenistan	0.02	0.02	0.02	0.04	0.03	0.05
Tuvalu	0.05	0.04	0.06	0.10	0.09	0.12
Uganda	0.02	0.02	0.02	0.03	0.03	0.04
Ukraine	0.02	0.02	0.03	0.04	0.04	0.05
United Arab Emirates	0.06	0.06	0.08	0.10	0.09	0.12
United Kingdom of Great Britain and Northern Ireland	0.03	0.03	0.04	0.07	0.06	0.08
United Republic of Tanzania	0.01	0.01	0.02	0.03	0.03	0.03
United States of America	0.04	0.04	0.05	0.10	0.10	0.13
United States Virgin Islands	0.08	0.07	0.09	0.14	0.13	0.17
Uruguay	0.03	0.03	0.04	0.07	0.06	0.08
Uzbekistan	0.02	0.02	0.02	0.06	0.05	0.07
Vanuatu	0.04	0.04	0.05	0.09	0.09	0.11
Yemen	0.03	0.03	0.04	0.06	0.06	0.08
Zambia	0.03	0.02	0.03	0.05	0.04	0.06
Zimbabwe	0.02	0.02	0.03	0.05	0.05	0.06

PAF = population attributable fraction.

Table S3. Global and regional burden of atrial fibrillation- versus diabetes-related ischaemic stroke: incidence, mortality, and disability-adjusted life years (1990 – 2021).

		**Incidence**					**DALY**					**Mortality**				
	**Disease**	**Cases (n), 1990**	**ASIR (per 100,000 population), 1990**	**Cases (n), 2021**	**ASIR (per 100,000 population), 2021**	**AAPC, 1990-2021**	**Cases (n), 1990**	**ASDR (per 100,000 population), 1990**	**Cases (n), 2021**	**ASDR (per 100,000 population), 2021**	**AAPC, 1990-2021**	**Cases (n), 1990**	**ASMR (per 100,000 population), 1990**	**Cases (n), 2021**	**ASMR (per 100,000 population), 2021**	**AAPC, 1990-2021**
Global	AF-IS	465460 (264071-768232)	14·97 (8·63-24)	973149 (567493-1576052)	12·11 (7·1-19)	-0·81 (-0·92 to -0·69)	6343235 (4485029-8530100)	208·65 (147·76-278)	10403409 (7174304-14248220)	132·29 (91·69-180)	-1·67 (-1·76 to -1·59)	424726 (299091-565311)	15·34 (11·17-20)	698979 (475126-954916)	9·18 (6·52-12)	-1·86 (-1·94 to -1·77)
	DM-IS	132989 (52298-268074)	3·75 (1·75-7)	462507 (189412-902097)	5·52 (2·47-10)	1·1 (1·02 to 1·18)	1645675 (830761-2663743)	47·92 (24·18-77)	4503161 (2248591-7302576)	54·74 (27·44-88)	0·17 (0·05 to 0·29)	90473 (45409-145652)	2·9 (2·2-6)	245242 (120654-398886)	3·1 (3·02-6)	-0·02 (-0·14 to 0·11)
Advanced Health System	AF-IS	305810 (178870-490816)	19·75 (11·64-31)	360655 (221459-557873)	11·09 (6·76-17)	-2·09 (-2·22 to -1·96)	3960761 (2872394-5112887)	258·19 (188·17-331)	3443068 (2388861-4582436)	103·7 (72·88-137)	-3·38 (-3·55 to -3·21)	279224 (199822-357541)	18·9 (13·87-24)	246535 (165407-326910)	7·0 (4·93-9)	-3·62 (-3·79 to -3·46)
	DM-IS	67743 (27391-133567)	4·25 (1·79-8)	143377 (61556-268475)	4·8 (2·12-9)	0·16 (0·03 to 0·29)	783489 (404683-1220827)	49·05 (25·36-76)	1240826 (623105-1963544)	39·98 (20·36-63)	-1·2 (-1·45 to -0·94)	46809 (23873-72516)	3·0 (2·3-6)	74431 (36233-117695)	2·21 (3·0-5)	-1·49 (-1·73 to -1·24)
Africa	AF-IS	17787 (9855-30238)	9·54 (5·42-15)	43314 (25054-70982)	10·04 (5·92-16)	0·16 (0·13 to 0·2)	274301 (176216-412061)	152·03 (98·14-225)	603180 (396956-890589)	145·55 (96·29-212)	-0·1 (-0·18 to -0·02)	17012 (10844-25471)	10·99 (7·38-16)	38393 (25112-56470)	10·54 (7·16-15)	-0·06 (-0·15 to 0·03)

	DM-IS	7583 (3008-15352)	3·38 (1·43-6)	35578 (14754-68704)	6·6 (2·85-12)	2·22 (2·18 to 2·26)	105376 (48802-186132)	50·32 (23·39-88)	437057 (209899-750824)	87·89 (42·48-149)	1·89 (1·8 to 1·98)	5661 (2607-10028)	3·17 (2·2-5)	22508 (10756-38699)	5·26 (3·55-9)	1·75 (1·65 to 1·84)
African Region	AF-IS	14448 (7886-24824)	9·67 (5·4-16)	33681 (19201-55634)	9·9 (5·74-16)	0·07 (0·04 to 0·1)	191005 (118786-300054)	135·44 (85·02-209)	424512 (275541-638743)	132·89 (87·06-196)	-0·1 (-0·19 to 0)	11514 (7060-18151)	9·59 (6·49-14)	26685 (17206-40227)	9·52 (6·64-14)	-0·04 (-0·14 to 0·06)
	DM-IS	5639 (2211-11522)	3·22 (1·35-6)	22121 (9013-43274)	5·32 (2·26-10)	1·66 (1·62 to 1·69)	67150 (30157-124151)	41·91 (18·94-76)	246626 (116296-434045)	65·37 (31·1-113)	1·44 (1·34 to 1·54)	3592 (1610-6713)	2·65 (1·97-5)	12797 (6004-22714)	3·92 (2·83-7)	1·29 (1·19 to 1·4)
America	AF-IS	86241 (48185-143090)	15·2 (8·57-25)	133456 (78738-212928)	9·81 (5·79-15)	-1·49 (-1·63 to -1·35)	835627 (599979-1088343)	149·79 (108·14-194)	1161586 (810058-1540670)	84·86 (59·74-112)	-2·06 (-2·18 to -1·94)	56950 (40015-73275)	10·58 (7·49-13)	80805 (54351-106065)	5·81 (4·16-7)	-2·16 (-2·29 to -2·02)
	DM-IS	22580 (8656-46328)	3·8 (1·55-7)	60817 (24921-118202)	4·51 (2·02-8)	0·5 (0·38 to 0·61)	189306 (96973-298159)	32·17 (16·53-50)	466836 (236112-738125)	34·29 (17·53-54)	0·03 (-0·09 to 0·15)	10446 (5281-16313)	1·83 (1·8-4)	26449 (12952-41517)	1·92 (2·24-4)	0·03 (-0·11 to 0·16)
Andean Latin America	AF-IS	1298 (752-2133)	7·75 (4·53-12)	3315 (1973-5279)	6·12 (3·66-9)	-0·78 (-0·9 to -0·66)	13990 (8965-20998)	83·61 (53·72-125)	28724 (17717-44783)	53·03 (32·74-82)	-1·63 (-1·82 to -1·45)	965 (613-1452)	6·02 (3·83-9)	2035 (1237-3201)	3·81 (2·72-5)	-1·63 (-1·81 to -1·45)
	DM-IS	312 (145-667)	1·7 (1·51-3)	1419 (600-2800)	2·51 (1·75-4)	1·18 (1·13 to 1·22)	3108 (1434-5629)	17·36 (7·96-31)	11461 (5083-21288)	20·55 (9·7-38)	0·29 (0·15 to 0·42)	179 (140-378)	1·06 (1·91-3)	678 (353-1358)	1·24 (1·8-3)	0·27 (0·13 to 0·41)
Asia	AF-IS	150330 (80974-259	11·13 (6·16-18)	515626 (290756-8	11·6 (6·61-19)	0·03 (-0·08	2076901 (1393615-	157·28 (105·84-224)	5554331 (3709492-	130·39 (87·53-184)	-0·63 (-0·72	127755 (85232-18	11·37 (7·95-16)	360312 (238128-5	9·09 (6·29-12)	-0·75 (-0·84

		591)		60900)		to 0·13)	3003699)		7924694)		to -0·55)	3425)		15457)		to -0·65)
	DM-IS	57902 (21938-120208)	3·44 (1·63-7)	277040 (110863-550919)	5·76 (2·63-11)	1·44 (1·35 to 1·53)	738159 (356165-1267701)	45·8 (22·15-78)	2708378 (1314982-4541351)	58·37 (28·57-97)	0·61 (0·51 to 0·71)	36982 (17706-63530)	2·71 (2·31-6)	142311 (68295-240620)	3·32 (2·83-7)	0·51 (0·38 to 0·63)
Australasia	AF-IS	3811 (2458-5594)	17·67 (11·45-25)	5875 (3642-8938)	9·65 (5·94-14)	-2·23 (-2·39 to -2·06)	36823 (25716-49587)	172·67 (121·57-230)	39006 (25810-53847)	60·57 (40·5-83)	-3·62 (-3·76 to -3·49)	2620 (1799-3527)	12·9 (9·12-17)	2928 (1867-4027)	4·29 (3·53-6)	-3·8 (-3·93 to -3·67)
	DM-IS	567 (278-1066)	2·49 (1·32-4)	1320 (595-2520)	2·33 (1·11-4)	-0·48 (-0·64 to -0·32)	4780 (2354-7927)	20·96 (10·26-34)	7274 (3406-12397)	11·97 (5·73-20)	-2·06 (-2·18 to -1·93)	295 (199-573)	1·35 (2·93-3)	455 (263-885)	0·69 (2·34-3)	-2·4 (-2·5 to -2·3)
Basic Health System	AF-IS	118717 (64487-203860)	11·86 (6·64-19)	460538 (259501-769782)	14·17 (8·08-23)	0·56 (0·5 to 0·62)	1725990 (1166907-2468729)	176·58 (119·96-248)	4846352 (3196698-6999277)	157·81 (104·73-226)	-0·32 (-0·47 to -0·18)	106822 (72149-151908)	12·81 (8·68-17)	316506 (206363-458719)	11·19 (7·7-16)	-0·39 (-0·55 to -0·22)
	DM-IS	47861 (18294-98837)	3·83 (1·85-7)	244279 (97858-485680)	6·87 (3·41-13)	1·78 (1·72 to 1·84)	643907 (313071-1090153)	53·68 (26·12-90)	2313017 (1105582-3929477)	68·02 (32·77-115)	0·7 (0·57 to 0·83)	32356 (15674-54909)	3·16 (2·79-6)	121727 (57290-209240)	3·92 (3·66-8)	0·67 (0·5 to 0·84)
Caribbean	AF-IS	2322 (1394-3679)	10·33 (6·26-16)	4850 (2951-7594)	8·94 (5·44-13)	-0·47 (-0·51 to -0·43)	33284 (23321-45565)	146·81 (102·97-199)	56710 (38043-81075)	104·91 (70·54-149)	-1·07 (-1·14 to -1·01)	2329 (1637-3149)	11·07 (7·77-14)	4157 (2783-5883)	7·58 (5·09-10)	-1·2 (-1·28 to -1·13)
	DM-IS	918	3·72	2918	5·41	1·15	12128	49·26	31548	58·42	0·54	711	3·07	1877	3·44 (2·5-6)	0·37

		(400-1811)	(1·73-7)	(1271-5508)	(2·45-10)	(1·13 to 1·17)	(6028-20061)	(24·51-81)	(15199-53929)	(28·32-99)	(0·47 to 0·6)	(410-1222)	(2·83-5)	(951-3239)		(0·29 to 0·44)
Central Africa	AF-IS	1683 (913-2918)	9·44 (5·26-15)	3577 (2020-5998)	8·92 (5·16-14)	-0·23 (-0·25 to -0·2)	22242 (12550-38005)	134·18 (77·28-223)	48287 (26982-84261)	130·24 (74·58-222)	-0·21 (-0·29 to -0·12)	1262 (697-2173)	9·25 (5·53-15)	2915 (1594-5145)	9·21 (5·47-15)	-0·13 (-0·22 to -0·05)
	DM-IS	696 (301-1461)	3·24 (1·35-6)	2585 (1063-5158)	4·87 (2·09-9)	1·32 (1·28 to 1·36)	8218 (3360-16527)	42·74 (17·73-84)	29526 (12044-60032)	64·37 (26·71-128)	1·29 (1·17 to 1·41)	415 (203-877)	2·63 (2·3-6)	1447 (606-3034)	3·78 (2·59-8)	1·13 (1·01 to 1·25)
Central Asia	AF-IS	5401 (3165-8699)	14·04 (8·28-22)	8933 (5319-14183)	14·76 (8·91-23)	0·25 (0·16 to 0·34)	73713 (51553-100142)	196·96 (138·05-265)	106543 (73683-147254)	181·59 (126·16-249)	-0·62 (-0·84 to -0·41)	4593 (3187-6221)	12·88 (9·28-17)	6568 (4524-9020)	12·12 (8·53-16)	-0·53 (-0·72 to -0·34)
	DM-IS	1356 (596-2683)	3·11 (1·96-6)	4843 (2083-9228)	6·56 (3·11-12)	2·61 (2·44 to 2·79)	16669 (8354-27080)	39·96 (20·22-64)	51229 (25609-83521)	73·5 (37·2-119)	1·7 (1·37 to 2·03)	841 (463-1432)	2·16 (2·3-5)	2481 (1288-4118)	4·0 (3·0-8)	1·74 (1·45 to 2·03)
Central Europe	AF-IS	28044 (16754-44381)	21·39 (12·9-33)	38929 (24073-59611)	16·31 (10·08-24)	-1·16 (-1·26 to -1·06)	489539 (359030-638214)	384·12 (281·32-499)	470359 (331160-629966)	200·21 (141·57-267)	-2·59 (-2·79 to -2·4)	32745 (23955-42443)	27·63 (20·32-35)	33130 (23059-44351)	14·03 (9·88-18)	-2·65 (-2·85 to -2·45)
	DM-IS	8970 (3831-17086)	6·41 (3·04-12)	15543 (6843-28491)	6·59 (3·23-12)	0·04 (-0·08 to 0·16)	142210 (74710-220338)	104·91 (55·03-162)	176349 (89482-279488)	74·53 (38·34-118)	-1·39 (-1·6 to -1·18)	8486 (4484-13155)	6·73 (4·92-11)	11053 (5607-17648)	4·57 (4·03-9)	-1·51 (-1·72 to -1·3)
Central Latin America	AF-IS	7357 (4237-1203	11·52 (6·73-18)	17592 (10458-27	7·78 (4·65-12)	-1·44 (-1·56	71201 (51920-93	112·63 (82·05-146)	136130 (93505-18	60·77 (41·93-83)	-2·13 (-2·28	4927 (3579-638	8·4 (6·08-10)	9540 (6450-132	4·31 (3·4-5)	-2·24 (-2·4 to

		8)		968)		to -1·31)	051)		8621)		to -1·98)	0)		06)		-2·09)
	DM-IS	3321 (1362-6682)	4·45 (2·35-8)	8676 (3559-169 75)	3·6 (1·87-7)	-1·07 (-1·24 to -0·91)	28019 (14687-43 624)	39·29 (20·77-61)	60804 (30105-10 0089)	25·76 (13·15-42)	-1·72 (-1·91 to -1·52)	1564 (847-2487)	2·4 (2·17-5)	3397 (1698-569 9)	1·48 (1·64-4)	-1·84 (-2·04 to -1·64)
Central Sub-Saharan Africa	AF-IS	1386 (749-2405)	10·84 (6·05-18)	3141 (1766-527 4)	9·83 (5·66-16)	-0·34 (-0·39 to -0·3)	16098 (8673-284 40)	136·03 (74·97-235)	37116 (19694-67 840)	127·11 (68·63-228)	-0·38 (-0·44 to -0·32)	872 (454-1562)	9·22 (5·17-16)	2206 (1131-410 8)	8·9 (4·85-16)	-0·28 (-0·34 to -0·21)
	DM-IS	620 (255-1304)	3·88 (1·73-7)	2379 (977-4745)	5·57 (2·46-10)	1·22 (1·18 to 1·26)	6422 (2512-133 40)	45·5 (18·11-92)	23400 (9101-497 22)	64·81 (25·67-135)	1·08 (1·03 to 1·12)	310 (143-695)	2·77 (2·88-6)	1122 (464-2488)	3·78 (2·49-9)	0·93 (0·88 to 0·98)
Commonwealth High Income	AF-IS	27187 (16153-430 20)	18·01 (10·77-28)	27370 (17208-41 376)	8·89 (5·57-13)	-2·52 (-2·68 to -2·36)	290507 (210170-3 75330)	193·97 (141·39-249)	185198 (125989-2 49374)	57·77 (39·74-77)	-4·22 (-4·42 to -4·03)	21019 (14941-26 974)	14·49 (10·52-18)	13350 (8767-176 86)	3·97 (3·16-5)	-4·48 (-4·69 to -4·28)
	DM-IS	5121 (2115-1002 2)	3·35 (1·47-6)	10220 (4515-187 63)	3·63 (1·68-6)	-0·08 (-0·22 to 0·06)	48093 (24586-75 574)	31·04 (15·9-48)	57464 (27905-93 412)	19·07 (9·4-31)	-2·04 (-2·22 to -1·85)	3048 (1573-481 1)	2·0 (2·52-4)	3453 (1680-557 2)	1·07 (1·85-3)	-2·48 (-2·68 to -2·28)
Commonwealth Low Income	AF-IS	5916 (3348-9839)	9·75 (5·64-15)	17557 (10178-28 673)	10·59 (6·26-17)	0·28 (0·23 to 0·33)	89286 (50912-14 9228)	146·04 (83·85-240)	253447 (145938-4 21256)	156·46 (90·84-257)	0·06 (-0·14 to 0·27)	5794 (3240-968 9)	10·62 (6·38-17)	17490 (9967-290 93)	12·02 (7·28-19)	0·17 (-0·1 to 0·43)
	DM-IS	1826	2·63	9350	4·67 (1·96-9)	1·95	24986	37·66	115851	61·8	1·58	1468	2·5 (2·05-4)	6604	3·95	1·36

		(733-3676)	(1·17-5)	(3815-18353)		(1·9 to 1·99)	(10234-49734)	(15·49-73)	(47729-228496)	(25·63-121)	(1·41 to 1·74)	(616-2952)		(2705-13128)	(2·76-8)	(1·13 to 1·59)
Commonwealth Middle Income	AF-IS	34813 (18372-60839)	8·91 (4·84-15)	87170 (48533-146914)	7·77 (4·4-12)	-0·65 (-0·74 to -0·57)	442683 (265238-706207)	110·34 (67·19-172)	1133076 (743644-1738017)	98·42 (65·24-147)	-0·4 (-0·49 to -0·31)	26704 (15857-42285)	7·77 (4·98-12)	72129 (47279-109032)	7·03 (4·9-10)	-0·29 (-0·42 to -0·17)
	DM-IS	12493 (4625-26449)	2·5 (1·31-5)	47240 (18406-96106)	3·4 (1·8-6)	0·77 (0·7 to 0·85)	145009 (61602-278780)	30·02 (13·03-56)	566610 (265092-1053029)	41·63 (19·75-76)	0·92 (0·85 to 0·99)	7226 (3046-13888)	1·77 (2·34-4)	28335 (13200-52275)	2·34 (2·88-5)	0·83 (0·73 to 0·92)
East Asia	AF-IS	59298 (30765-105220)	10·67 (5·72-18)	304210 (166973-518848)	15·38 (8·53-25)	1·2 (1·1 to 1·29)	865035 (554023-1331794)	168·03 (108·83-253)	2949589 (1844017-4474637)	164·97 (104·2-247)	0·01 (-0·24 to 0·26)	50589 (32089-78007)	12·33 (8·26-18)	190642 (117361-291806)	11·88 (7·64-17)	-0·07 (-0·37 to 0·22)
	DM-IS	25969 (9569-55001)	3·54 (2·08-7)	144185 (56301-292757)	6·74 (3·41-13)	1·86 (1·74 to 1·97)	356985 (164235-646968)	51·31 (23·89-92)	1244116 (560410-2230498)	61·86 (28·37-110)	0·43 (0·22 to 0·64)	16784 (7645-30775)	2·96 (2·37-6)	64240 (28305-117365)	3·54 (2·78-8)	0·41 (0·14 to 0·69)
East Asia & Pacific - WB	AF-IS	109987 (58684-191311)	12·05 (6·6-20)	410057 (229390-688683)	13·2 (7·44-22)	0·22 (0·08 to 0·35)	1517692 (1012929-2203503)	175·2 (117·71-249)	4125468 (2660805-6060339)	139·61 (90·75-203)	-0·71 (-0·83 to -0·6)	92952 (61797-134257)	12·75 (8·53-18)	265878 (168391-392923)	9·53 (6·39-13)	-0·93 (-1·06 to -0·8)
	DM-IS	43506 (16434-90620)	3·87 (2·23-8)	213860 (85373-425846)	6·62 (3·15-13)	1·47 (1·38 to 1·57)	553306 (265491-952062)	51·99 (25·15-88)	1952830 (913208-3374076)	62·64 (29·84-107)	0·43 (0·3 to 0·57)	27657 (13215-47761)	3·09 (2·83-6)	104307 (47799-182347)	3·56 (2·67-7)	0·3 (0·13 to 0·48)
Eastern Africa	AF-IS	4050 (2213-6981)	9·09 (5·11-15)	10277 (5816-17056)	9·61 (5·54-15)	0·2 (0·16 to 0·25)	47281 (28678-77426)	110·89 (67·56-177)	104198 (63366-164411)	101·56 (61·85-158)	-0·47 (-0·51 to -0·42)	2723 (1619-4490)	7·7 (5·07-12)	6341 (3759-10099)	7·03 (4·58-11)	-0·46 (-0·51 to -0·41)

	DM-IS	1451 (580-2990)	2·77 (1·14-5)	5282 (2146-10430)	4·17 (1·74-8)	1·3 (1·26 to 1·34)	15525 (6822-29934)	32·13 (14·13-61)	48676 (21614-89951)	41·64 (18·56-76)	0·61 (0·56 to 0·67)	809 (376-1604)	2·02 (2·33-4)	2525 (1117-4753)	2·5 (2·62-5)	0·5 (0·44 to 0·56)
Eastern Europe	AF-IS	56948 (30393-99036)	24·44 (13·22-42)	65456 (36981-108985)	18·46 (10·39-30)	-0·95 (-1·06 to -0·84)	961137 (699994-1254700)	416·87 (304·11-542)	846957 (583004-1167092)	246·03 (170·05-338)	-2·4 (-2·85 to -1·95)	63210 (45817-82085)	29·56 (21·69-38)	58375 (39820-80273)	16·77 (11·68-22)	-2·57 (-3·03 to -2·12)
	DM-IS	11245 (4273-23504)	4·5 (2·01-9)	20953 (8410-41810)	5·89 (2·64-11)	0·72 (0·56 to 0·88)	177648 (92646-276123)	72·25 (38·19-112)	259041 (128235-422932)	74·2 (37·17-121)	-0·78 (-1·3 to -0·25)	10419 (5449-16209)	4·56 (3·86-8)	15625 (7697-25651)	4·41 (3·84-9)	-1·03 (-1·56 to -0·5)
Eastern Mediterranean Region	AF-IS	12050 (6810-20118)	10·1 (5·84-16)	29502 (17230-47667)	9·9 (5·88-15)	-0·04 (-0·08 to -0·01)	216819 (135163-326774)	177·47 (110·91-265)	456171 (299164-663846)	153·3 (100·81-221)	-0·51 (-0·56 to -0·47)	14239 (8828-21353)	13·18 (8·18-19)	30389 (19900-43913)	11·36 (7·8-16)	-0·49 (-0·54 to -0·45)
	DM-IS	6002 (2392-12106)	3·98 (1·76-7)	33868 (14194-64383)	8·67 (3·96-16)	2·68 (2·57 to 2·79)	101288 (45680-180051)	69·09 (31·18-122)	462335 (222833-781561)	126·2 (61·06-211)	2·02 (1·91 to 2·13)	5264 (2385-9391)	4·14 (2·94-7)	23242 (11164-39175)	7·35 (4·51-13)	1·96 (1·86 to 2·07)
Eastern Sub-Saharan Africa	AF-IS	4400 (2382-7630)	9·1 (5·06-15)	10816 (6098-18075)	9·62 (5·52-15)	0·22 (0·14 to 0·3)	46859 (27319-79003)	103·51 (60·93-171)	108223 (65231-174020)	101·15 (61·3-160)	-0·17 (-0·22 to -0·13)	2633 (1494-4499)	7·01 (4·34-11)	6370 (3727-10399)	6·82 (4·34-10)	-0·17 (-0·21 to -0·13)
	DM-IS	1373 (548-2837)	2·52 (1·04-5)	4659 (1888-9268)	3·54 (1·47-6)	1·12 (1·04 to 1·19)	13506 (5703-26724)	27·5 (11·68-53)	42859 (18866-80578)	35·67 (15·84-66)	0·73 (0·69 to 0·76)	712 (316-1457)	1·74 (2·62-4)	2201 (976-4240)	2·12 (2·78-5)	0·54 (0·5 to 0·57)
Europe	AF-IS	211832	21·48	233604	12·89	-1·89	3068187	314·41	2423971	132·66	-3·28	217290	23·19	175870	9·14	-3·48

		(126242-334896)	(12·9-33)	(144971-357233)	(7·94-19)	(-1·99 to -1·78)	(2235224-3952212)	(229·89-403)	(1699030-3212879)	(93·94-175)	(-3·48 to -3·07)	(156859-278175)	(17·01-29)	(120454-232564)	(6·43-12)	(-3·67 to -3·29)
	DM-IS	41830 (17260-81193)	4·11 (1·89-8)	74038 (32048-137671)	4·4 (2·14-8)	0·03 (-0·07 to 0·13)	553658 (286592-860574)	54·41 (28·25-84)	714333 (359323-1132874)	41·31 (21·1-65)	-1·44 (-1·7 to -1·18)	33946 (17408-52622)	3·43 (2·95-6)	44009 (21664-69939)	2·37 (2·48-5)	-1·7 (-1·94 to -1·46)
Europe & Central Asia - WB	AF-IS	215240 (128374-340243)	21·3 (12·8-33)	240205 (149125-367087)	13·0 (8·02-19)	-1·83 (-1·93 to -1·72)	3113395 (2267136-4012217)	311·29 (227·48-399)	2497971 (1753371-3311392)	134·38 (95·28-177)	-3·2 (-3·41 to -2·99)	219883 (158623-281595)	22·89 (16·79-29)	180121 (123665-238116)	9·22 (6·52-12)	-3·41 (-3·6 to -3·22)
	DM-IS	42667 (17624-82754)	4·08 (1·87-7)	77401 (33531-143744)	4·49 (2·14-8)	0·12 (0·02 to 0·22)	563878 (291818-876529)	54·05 (28·05-84)	748376 (377244-1186276)	42·39 (21·78-67)	-1·33 (-1·59 to -1·07)	34408 (17640-53333)	3·39 (2·89-6)	45543 (22498-72306)	2·41 (2·75-5)	-1·61 (-1·85 to -1·37)
European Region	AF-IS	216656 (129268-342321)	21·3 (12·8-33)	242827 (150786-370947)	13·01 (8·03-19)	-1·82 (-1·92 to -1·72)	3130202 (2279570-4033630)	310·89 (227·19-399)	2527178 (1774796-3348616)	134·66 (95·51-178)	-3·19 (-3·4 to -2·98)	220993 (159435-282971)	22·85 (16·77-29)	182145 (125111-240651)	9·24 (6·54-12)	-3·39 (-3·58 to -3·2)
	DM-IS	43057 (17790-83478)	4·09 (1·88-7)	78219 (33885-145230)	4·49 (2·14-8)	0·11 (0·02 to 0·21)	568368 (294181-883454)	54·12 (28·08-84)	756762 (381599-1199177)	42·44 (21·81-67)	-1·33 (-1·59 to -1·07)	34667 (17775-53724)	3·39 (2·89-6)	46031 (22748-73043)	2·41 (2·73-5)	-1·6 (-1·85 to -1·36)
High-income Asia Pacific	AF-IS	23359 (12366-409	13·77 (7·36-23)	31600 (18402-51	5·66 (3·25-9)	-3·33 (-3·71	256733 (175537-3	159·65 (109·53-216)	258731 (162741-3	41·97 (27·19-59)	-4·59 (-4·87	17454 (11669-23	11·72 (7·87-15)	18376 (10793-26	2·62 (1·94-3)	-5·1 (-5·38

		67)		325)		to -2·95)	49763)		71890)		to -4·31)	678)		468)		to -4·82)
	DM-IS	10106 (3855-21060)	5·56 (2·5-11)	21258 (8954-40481)	4·46 (1·98-8)	-1·51 (-1·79 to -1·23)	92403 (46188-148316)	54·03 (27·2-86)	147386 (68822-245360)	26·78 (12·9-44)	-2·93 (-3·16 to -2·7)	5286 (2635-8521)	3·37 (3·89-6)	8938 (3968-14968)	1·36 (2·74-3)	-3·59 (-3·82 to -3·36)
High-income North America	AF-IS	59065 (32456-99264)	16·52 (9·13-27)	76013 (44538-121589)	10·6 (6·2-16)	-1·45 (-1·63 to -1·27)	468775 (330005-618398)	132·53 (94·51-173)	636007 (434249-849279)	86·27 (59·63-114)	-1·89 (-2·14 to -1·65)	32047 (21924-41576)	9·16 (6·53-11)	43498 (28291-57101)	5·65 (4·19-7)	-2·13 (-2·42 to -1·85)
	DM-IS	12536 (4693-26259)	3·55 (1·38-7)	33975 (13778-66468)	5·01 (2·06-9)	1·18 (1·02 to 1·34)	83126 (41191-134776)	23·22 (11·57-37)	241965 (118414-390489)	34·43 (16·96-55)	0·91 (0·63 to 1·18)	4618 (2258-7367)	1·29 (1·93-3)	13299 (6277-21065)	1·79 (2·81-4)	0·63 (0·3 to 0·97)
Latin America & Caribbean - WB	AF-IS	26986 (15363-44521)	12·73 (7·36-20)	55190 (32572-88664)	8·44 (5·0-13)	-1·5 (-1·63 to -1·38)	338989 (245093-442635)	161·92 (116·84-210)	492427 (344189-660510)	75·88 (53·32-101)	-2·43 (-2·54 to -2·32)	22834 (16370-29641)	11·82 (8·44-15)	34559 (23617-46270)	5·37 (3·94-7)	-2·47 (-2·58 to -2·36)
	DM-IS	10233 (4056-20593)	4·21 (2·07-8)	26312 (10812-51264)	3·86 (2·24-7)	-0·49 (-0·61 to -0·37)	112009 (58433-173730)	47·82 (24·92-74)	212781 (108508-336371)	31·59 (16·66-49)	-1·38 (-1·49 to -1·26)	6242 (3264-9704)	2·88 (2·41-5)	12225 (6121-19435)	1·85 (2·4-4)	-1·4 (-1·5 to -1·3)
Limited Health System	AF-IS	44489 (24102-76591)	8·96 (5·0-15)	115826 (65927-191077)	8·21 (4·76-13)	-0·44 (-0·51 to -0·37)	610681 (374348-961514)	120·31 (74·37-185)	1558366 (1027142-2368035)	108·89 (72·36-162)	-0·42 (-0·5 to -0·35)	36895 (22418-57798)	8·5 (5·56-13)	100357 (65912-150961)	7·87 (5·53-11)	-0·33 (-0·43 to -0·23)

	DM-IS	15875 (6012-33088)	2·5 (1·22-5)	63465 (25270-126386)	3·63 (1·66-7)	1·05 (0·99 to 1·11)	197044 (86333-371588)	32·26 (14·23-60)	772250 (363851-1410161)	45·6 (21·61-82)	0·97 (0·9 to 1·03)	9873 (4262-18649)	1·91 (2·56-4)	39186 (18328-71196)	2·61 (2·88-5)	0·88 (0·81 to 0·96)
Middle East & North Africa - WB	AF-IS	8072 (4671-13252)	10·4 (6·16-16)	24340 (14505-38843)	10·19 (6·17-16)	-0·04 (-0·08 to 0)	156767 (99472-236069)	198·64 (126·61-295)	378255 (246961-554891)	160·29 (104·96-233)	-0·7 (-0·76 to -0·64)	10544 (6686-15776)	15·09 (9·6-22)	26107 (16975-38045)	12·1 (7·87-17)	-0·69 (-0·76 to -0·63)
	DM-IS	4750 (1959-9399)	4·73 (2·1-9)	29557 (12662-55498)	9·74 (4·46-18)	2·44 (2·39 to 2·5)	86331 (39504-153563)	88·79 (40·74-156)	406185 (195203-690558)	141·53 (68·31-238)	1·55 (1·5 to 1·59)	4557 (2104-8119)	5·45 (3·79-9)	20859 (9986-35428)	8·28 (4·77-14)	1·41 (1·35 to 1·46)
Minimal Health System	AF-IS	3377 (1864-5765)	8·76 (4·97-14)	6423 (3666-10669)	8·35 (4·9-13)	-0·16 (-0·18 to -0·13)	50351 (29982-82618)	134·57 (80·75-217)	95519 (57273-157438)	130·75 (79·71-211)	-0·07 (-0·11 to -0·03)	2942 (1721-4845)	9·38 (5·91-15)	5795 (3430-9630)	9·27 (6·04-15)	0 (-0·03 to 0·04)
	DM-IS	1601 (637-3266)	3·4 (1·45-6)	5572 (2277-10956)	5·47 (2·34-10)	1·55 (1·53 to 1·56)	21346 (9212-41158)	48·91 (21·2-93)	70156 (30687-134083)	78·18 (34·72-147)	1·58 (1·5 to 1·65)	1093 (484-2144)	3·0 (2·02-6)	3477 (1512-6757)	4·62 (3·12-9)	1·47 (1·41 to 1·54)
North Africa and Middle East	AF-IS	10274 (5917-16979)	9·5 (5·59-15)	28104 (16647-45186)	8·73 (5·24-13)	-0·35 (-0·4 to -0·31)	196360 (124134-292591)	178·65 (113·1-263)	427175 (278000-623839)	134·56 (87·7-195)	-0·99 (-1·05 to -0·92)	13246 (8327-19658)	13·54 (8·51-19)	29917 (19341-43612)	10·25 (6·99-14)	-0·93 (-1 to -0·85)
	DM-IS	6375 (2612-12642)	4·59 (2·02-8)	36490 (15589-68599)	9·08 (4·14-16)	2·27 (2·23 to 2·31)	113152 (51988-197731)	84·28 (38·74-146)	490206 (236029-824921)	128·43 (62·03-214)	1·4 (1·35 to 1·44)	6004 (2767-10520)	5·15 (3·56-9)	25667 (12283-43262)	7·61 (4·48-13)	1·33 (1·28 to 1·39)
North	AF-IS	59063	16·51	76015	10·6 (6·2-16)	-1·45	468783	132·52	636038	86·26	-1·89	32049	9·16	43502	5·65	-2·13

America		(32455-99260)	(9·13-27)	(44539-121591)		(-1·63 to -1·27)	(330014-618406)	(94·5-173)	(434271-849315)	(59·63-114)	(-2·14 to -1·65)	(21925-41577)	(6·53-11)	(28294-57106)	(4·19-7)	(-2·42 to -1·85)
	DM-IS	12536 (4693-26259)	3·55 (1·38-7)	33975 (13778-66468)	5·01 (2·06-9)	1·18 (1·02 to 1·34)	83132 (41195-134785)	23·22 (11·57-37)	241976 (118420-390504)	34·43 (16·96-55)	0·9 (0·63 to 1·18)	4618 (2259-7367)	1·29 (1·93-3)	13300 (6278-21067)	1·79 (2·81-4)	0·63 (0·3 to 0·97)
Northern Africa	AF-IS	3633 (2108-5999)	9·42 (5·61-15)	10848 (6357-17484)	10·36 (6·2-16)	0·24 (0·19 to 0·29)	80521 (47004-130628)	210·35 (123·65-336)	186205 (113955-292253)	184·59 (113·52-286)	-0·18 (-0·26 to -0·1)	5389 (3156-8668)	16·3 (9·58-25)	12665 (7742-19745)	14·21 (8·7-22)	-0·11 (-0·21 to -0·01)
	DM-IS	2177 (911-4319)	4·34 (1·95-8)	16188 (6907-30313)	12·02 (5·41-22)	3·36 (3·31 to 3·42)	44284 (18431-85811)	93·05 (39·04-177)	241883 (108780-438207)	192·85 (87·42-345)	2·63 (2·52 to 2·75)	2382 (1017-4603)	5·9 (3·65-11)	12581 (5696-22729)	11·77 (6·1-21)	2·6 (2·47 to 2·72)
Oceania	AF-IS	152 (84-260)	9·36 (5·36-15)	390 (223-643)	8·51 (5·0-13)	-0·38 (-0·41 to -0·35)	1901 (1072-3353)	128·07 (74·77-217)	4685 (2736-7980)	111·96 (66·42-186)	-0·53 (-0·59 to -0·47)	103 (57-185)	8·79 (5·47-15)	271 (153-470)	7·6 (4·73-12)	-0·57 (-0·64 to -0·51)
	DM-IS	98 (87-258)	4·5 (1·99-9)	454 (239-933)	7·25 (3·22-13)	1·35 (1·24 to 1·46)	1089 (474-2317)	56·05 (23·26-113)	4563 (1971-9077)	82·14 (35·47-160)	1·07 (0·96 to 1·17)	48 (83-184)	3·24 (2·35-7)	198 (135-478)	4·4 (2·92-10)	0·83 (0·75 to 0·92)
Region of the Americas	AF-IS	86241 (48185-143090)	15·2 (8·57-25)	133456 (78738-212928)	9·81 (5·79-15)	-1·49 (-1·63 to -1·35)	835627 (599979-1088343)	149·79 (108·14-194)	1161586 (810058-1540670)	84·86 (59·74-112)	-2·06 (-2·18 to -1·94)	56950 (40015-73275)	10·58 (7·49-13)	80805 (54351-106065)	5·81 (4·16-7)	-2·16 (-2·29 to -2·02)
	DM-IS	22580	3·8 (1·55-7)	60817	4·51 (2·02-8)	0·5	189306	32·17	466836	34·29	0·03	10446	1·83 (1·8-4)	26449	1·92	0·03

		(8656-46328)		(24921-118202)		(0·38 to 0·61)	(96973-298159)	(16·53-50)	(236112-738125)	(17·53-54)	(-0·09 to 0·15)	(5281-16313)		(12952-41517)	(2·24-4)	(-0·11 to 0·16)
South Asia	AF-IS	29570 (15694-51479)	8·42 (4·62-14)	79156 (44414-132490)	7·45 (4·26-12)	-0·63 (-0·74 to -0·53)	385073 (223596-634535)	104·4 (61·48-168)	1070754 (681547-1705537)	97·15 (62·48-151)	-0·35 (-0·46 to -0·23)	23332 (13402-38129)	7·4 (4·54-11)	69681 (44242-109615)	7·09 (4·74-11)	-0·24 (-0·39 to -0·09)
	DM-IS	10288 (3796-21831)	2·23 (1·38-4)	40849 (15910-83131)	3·08 (1·46-6)	0·83 (0·74 to 0·92)	122107 (50186-243460)	27·18 (11·41-53)	506511 (228676-986418)	38·77 (17·81-74)	0·98 (0·88 to 1·08)	6041 (2455-12046)	1·59 (1·98-4)	25654 (11529-49616)	2·2 (2·33-5)	0·89 (0·76 to 1·02)
South Asia - WB	AF-IS	31114 (16634-53911)	8·58 (4·74-14)	82527 (46536-137542)	7·57 (4·36-12)	-0·64 (-0·74 to -0·54)	415426 (244664-675988)	109·53 (65·49-174)	1127672 (721303-1777349)	100·03 (64·62-154)	-0·38 (-0·48 to -0·27)	25343 (14841-40840)	7·81 (4·89-12)	73686 (47136-114630)	7·33 (4·95-11)	-0·27 (-0·41 to -0·13)
	DM-IS	11112 (4132-23446)	2·35 (1·44-4)	44010 (17248-89077)	3·25 (1·57-6)	0·83 (0·74 to 0·91)	135306 (56430-266337)	29·38 (12·52-56)	550737 (250303-1059907)	41·28 (19·07-78)	0·94 (0·85 to 1·03)	6764 (2805-13290)	1·74 (2·18-4)	28115 (12753-53673)	2·36 (2·63-5)	0·86 (0·75 to 0·97)
South-East Asia Region	AF-IS	42083 (22772-72470)	9·7 (5·41-16)	120396 (68347-199502)	8·87 (5·13-14)	-0·46 (-0·53 to -0·39)	567380 (355754-878910)	128·38 (81·33-195)	1623640 (1072489-2394597)	118·58 (79·01-172)	-0·33 (-0·41 to -0·25)	34251 (21361-52848)	9·06 (6·15-13)	104515 (69039-152878)	8·49 (5·99-12)	-0·25 (-0·36 to -0·15)
	DM-IS	14328 (5410-29969)	2·57 (1·56-5)	63430 (25194-126708)	3·92 (2·11-7)	1·19 (1·12 to 1·25)	176072 (78495-327301)	32·93 (14·91-60)	786369 (370092-1385622)	49·74 (23·71-86)	1·22 (1·16 to 1·28)	8724 (3860-16304)	1·94 (2·67-5)	40442 (18975-71189)	2·87 (2·73-6)	1·2 (1·13 to 1·27)
Southeast Asia	AF-IS	24220 (13535-407	13·73 (7·83-22)	70193 (40993-11	13·95 (8·28-22)	0·05 (0 to 0·1)	356460 (231288-5	207·23 (135·19-302)	981718 (628766-1	204·15 (132·09-295)	0·04 (-0·11	22254 (14304-32	14·59 (9·42-21)	62760 (40067-91	14·25 (9·18-20)	0·05 (-0·13

		14)		3640)			25437)		436654)		to 0·2)	889)		549)		to 0·23)
	DM-IS	7673 (3047-15571)	3·8 (2·06-7)	40179 (16771-76995)	7·2 (4·08-13)	2·12 (2·09 to 2·16)	105397 (48955-184602)	54·86 (25·75-95)	518650 (238989-893754)	98·78 (46·04-168)	2·08 (1·93 to 2·22)	5701 (2643-10120)	3·38 (3·0-7)	28394 (13008-49130)	6·0 (4·12-11)	2·1 (1·91 to 2·29)
Southern Africa	AF-IS	3838 (2100-6576)	12·26 (6·81-20)	8699 (4912-14546)	13·37 (7·68-21)	0·27 (0·11 to 0·43)	37981 (24362-56627)	129·51 (83·19-191)	95343 (63389-138380)	155·6 (104·37-222)	0·68 (0·38 to 0·98)	2310 (1447-3478)	8·92 (5·95-13)	5871 (3884-8539)	10·89 (7·77-15)	0·77 (0·45 to 1·09)
	DM-IS	1372 (551-2822)	3·91 (1·62-7)	5790 (2372-11419)	7·35 (3·14-14)	2·2 (2·04 to 2·37)	12470 (5784-21937)	38·65 (17·87-67)	57561 (27796-97806)	79·33 (38·55-133)	2·59 (2·3 to 2·88)	678 (339-1242)	2·41 (2·95-5)	2959 (1467-5105)	4·66 (3·42-9)	2·42 (2·1 to 2·74)
Southern Latin America	AF-IS	4074 (2392-6613)	9·95 (5·88-16)	4613 (2720-7363)	5·25 (3·09-8)	-2·32 (-2·6 to -2·05)	55040 (37934-75604)	138·65 (95·79-189)	41258 (27726-58021)	47·15 (31·95-66)	-3·22 (-3·47 to -2·97)	3786 (2589-5181)	10·15 (7·41-14)	2879 (1885-4052)	3·26 (2·94-4)	-3·31 (-3·58 to -3·04)
	DM-IS	1451 (634-2883)	3·24 (1·5-6)	3471 (1494-6581)	3·94 (1·85-7)	0·38 (0·22 to 0·55)	16174 (8004-26490)	37·12 (18·39-60)	26481 (13135-43255)	29·79 (14·9-48)	-0·53 (-0·67 to -0·39)	923 (490-1565)	2·23 (2·77-5)	1528 (827-2583)	1·69 (2·56-4)	-0·62 (-0·78 to -0·46)
Southern Sub-Saharan Africa	AF-IS	2774 (1493-4810)	13·11 (7·13-22)	6083 (3358-10294)	14·3 (8·01-23)	0·23 (0 to 0·47)	24753 (15633-36160)	125·72 (79·93-181)	61504 (41890-86553)	152·96 (104·75-213)	0·74 (0·31 to 1·16)	1560 (960-2296)	8·74 (5·44-12)	3924 (2648-5509)	10·88 (7·91-15)	0·86 (0·41 to 1·31)
	DM-IS	987 (406-2073)	4·19 (1·77-8)	4133 (1676-8272)	8·11 (3·41-16)	2·29 (2·04 to 2·53)	8008 (3647-13896)	36·77 (16·81-63)	37790 (18814-61958)	79·14 (39·47-129)	2·8 (2·39 to 3·22)	442 (219-839)	2·26 (3·06-5)	1996 (1011-3339)	4·66 (3·46-9)	2·69 (2·23 to 3·15)
Sub-Saharan	AF-IS	14211	9·53	32396	9·82	0·12	190379	134·09	411611	131·44	-0·1	11429	9·44	25463	9·27	-0·08

Africa - WB		(7727-24430)	(5·3-16)	(18365-53756)	(5·67-16)	(0·08 to 0·16)	(117637-301648)	(83·49-208)	(266484-621579)	(86·06-195)	(-0·2 to 0)	(6928-18229)	(6·31-14)	(16328-38595)	(6·48-13)	(-0·18 to 0·02)
	DM-IS	5415 (2117-11078)	3·11 (1·31-6)	20097 (8138-39555)	4·97 (2·1-9)	1·56 (1·51 to 1·61)	65580 (29289-122343)	40·9 (18·35-75)	227975 (106993-402892)	61·9 (29·36-108)	1·34 (1·23 to 1·45)	3501 (1547-6624)	2·57 (1·95-5)	11696 (5457-20891)	3·67 (2·87-7)	1·17 (1·05 to 1·29)
Tropical Latin America	AF-IS	12264 (6435-21451)	18·03 (9·69-30)	25100 (13930-41887)	10·8 (6·03-17)	-1·86 (-1·96 to -1·75)	170313 (121966-223485)	257·56 (183·7-336)	232852 (160002-311799)	101·57 (70·29-135)	-2·94 (-3·04 to -2·83)	11110 (7874-14514)	18·83 (13·26-24)	16162 (10784-21691)	7·18 (5·18-9)	-2·98 (-3·09 to -2·86)
	DM-IS	4114 (1560-8758)	5·08 (2·27-10)	9757 (3837-19718)	4·0 (2·19-8)	-0·79 (-0·91 to -0·67)	50192 (25890-78488)	65·41 (33·54-102)	83020 (41639-131576)	34·63 (18·07-54)	-1·84 (-1·99 to -1·69)	2735 (1440-4339)	3·97 (2·86-7)	4783 (2358-7712)	2·05 (2·6-4)	-1·8 (-1·96 to -1·65)
Western Africa	AF-IS	4651 (2491-8085)	8·39 (4·59-14)	9908 (5563-16520)	8·5 (4·86-13)	0·13 (0·1 to 0·17)	81177 (46431-135906)	151·5 (88·06-248)	162805 (100083-255757)	145·31 (90·64-224)	-0·11 (-0·17 to -0·04)	5010 (2815-8419)	10·79 (6·61-17)	10264 (6261-16150)	10·35 (6·86-16)	-0·11 (-0·17 to -0·04)
	DM-IS	1886 (746-3922)	2·9 (1·29-5)	6580 (2650-13059)	4·5 (1·99-8)	1·47 (1·43 to 1·51)	29158 (12041-57651)	48·13 (20·09-93)	94600 (41862-174432)	71·49 (32·05-130)	1·28 (1·2 to 1·36)	1577 (698-3173)	3·02 (2·27-6)	4877 (2190-9098)	4·23 (2·71-8)	1·11 (1·03 to 1·18)
Western Europe	AF-IS	116687 (72454-177486)	19·62 (12·21-29)	113154 (72896-166530)	9·95 (6·34-14)	-2·5 (-2·62 to -2·37)	1383094 (991538-1789478)	235·25 (169·9-303)	837485 (563525-1127056)	69·02 (47·19-92)	-4·23 (-4·4 to -4·06)	103404 (72873-133137)	18·08 (13·01-23)	62576 (40324-83775)	4·79 (3·86-6)	-4·56 (-4·73 to -4·39)
	DM-IS	19642	3·32 (1·5-6)	30649	3·05 (1·45-5)	-0·58	201501	33·54	191824	17·23	-2·48	13332	2·24	12398	1·0 (2·09-3)	-2·95

		(8388-36986)		(13724-55155)		(-0·69 to -0·47)	(101485-318865)	(17·01-53)	(91524-313865)	(8·49-28)	(-2·65 to -2·31)	(6608-21093)	(2·21-4)	(5705-20238)		(-3·11 to -2·79)
Western Pacific Region	AF-IS	91321 (48296-159807)	11·65 (6·33-19)	354490 (197379-598049)	12·98 (7·28-21)	0·28 (0·11 to 0·45)	1271882 (838221-1869683)	172·28 (114·5-248)	3470987 (2200577-5164724)	134·09 (85·73-198)	-0·77 (-0·89 to -0·65)	78329 (51363-114476)	12·7 (8·76-18)	225601 (140477-339095)	9·21 (6·04-13)	-1 (-1·13 to -0·87)
	DM-IS	37829 (14178-79178)	3·9 (2·19-8)	183985 (73136-367892)	6·54 (3·08-13)	1·38 (1·26 to 1·49)	485903 (229901-847950)	53·09 (25·4-91)	1623042 (745371-2842305)	59·84 (27·95-104)	0·19 (0·05 to 0·33)	24374 (11480-42688)	3·19 (2·7-7)	87285 (39237-155496)	3·41 (2·29-7)	0·02 (-0·15 to 0·19)
Western Sub-Saharan Africa	AF-IS	5084 (2730-8808)	8·28 (4·54-14)	10966 (6183-18235)	8·44 (4·84-13)	0·15 (0·11 to 0·18)	88594 (51025-147861)	149·21 (87·31-243)	182254 (112626-286167)	145·69 (91·32-225)	-0·05 (-0·13 to 0·03)	5465 (3092-9145)	10·62 (6·55-17)	11465 (7031-18031)	10·38 (6·55-16)	-0·05 (-0·12 to 0·03)
	DM-IS	2059 (808-4268)	2·86 (1·28-5)	7339 (2956-14527)	4·48 (2·0-8)	1·51 (1·46 to 1·55)	31772 (13212-62623)	47·34 (19·9-91)	106420 (47380-196144)	71·8 (32·38-130)	1·36 (1·26 to 1·46)	1718 (747-3439)	2·96 (2·25-6)	5472 (2454-10206)	4·24 (2·69-8)	1·18 (1·08 to 1·28)
SDI															
High SDI	AF-IS	187595 (111160-298069)	17·52 (10·43-27)	241622 (147484-375952)	10·1 (6·12-15)	-2·04 (-2·18 to -1·9)	2084965 (1493507-2713872)	197·93 (142·87-256)	1931258 (1309082-2616902)	77·99 (53·68-105)	-3·33 (-3·49 to -3·18)	147789 (103965-190531)	14·46 (10·52-18)	133771 (86517-179703)	5·07 (3·49-6)	-3·72 (-3·88 to -3·56)
	DM-IS	41223 (16814-80752)	3·79 (1·65-7)	99240 (42346-187113)	4·55 (2·02-8)	0·31 (0·17 to 0·45)	392087 (199312-619359)	36·09 (18·4-56)	687263 (335708-1111916)	29·88 (14·83-48)	-0·97 (-1·15 to -0·8)	23397 (11719-36676)	2·19 (2·59-4)	39497 (18522-63474)	1·57 (2·74-4)	-1·46 (-1·64 to -1·27)

High-middle SDI	AF-IS	148040 (83005-246981)	18·71 (10·68-30)	283511 (164439-462918)	14·8 (8·61-24)	-0·87 (-1·01 to -0·73)	2273505 (1622736-3016991)	294·76 (210·29-389)	3083374 (2089248-4311834)	165·42 (112·56-230)	-2·2 (-2·36 to -2·04)	153533 (109299-201926)	22·08 (16·04-28)	215034 (143769-300165)	11·85 (8·17-16)	-2·34 (-2·5 to -2·19)
	DM-IS	42162 (16536-85404)	4·72 (2·27-9)	124360 (50750-244193)	6·36 (2·98-12)	0·8 (0·72 to 0·88)	592457 (302701-940340)	68·27 (34·78-108)	1220372 (597322-2023369)	63·38 (31·29-105)	-0·7 (-0·97 to -0·43)	33374 (17012-52726)	4·26 (3·53-8)	69412 (33457-115619)	3·71 (3·13-7)	-0·88 (-1·15 to -0·61)
Middle SDI	AF-IS	77863 (42007-134055)	11·41 (6·34-19)	292744 (165428-486750)	12·9 (7·39-21)	0·36 (0·31 to 0·4)	1105550 (749174-1594884)	164·91 (112·3-234)	3114483 (2086530-4439826)	143·78 (96·76-203)	-0·47 (-0·56 to -0·37)	68317 (46158-97789)	11·95 (8·1-16)	203015 (134740-289710)	10·19 (7·06-14)	-0·53 (-0·65 to -0·42)
	DM-IS	31059 (11797-64423)	3·63 (1·76-7)	151907 (60801-301599)	6·05 (2·81-12)	1·52 (1·46 to 1·59)	406811 (198295-694705)	49·65 (24·22-84)	1466432 (712197-2462144)	60·83 (29·76-101)	0·54 (0·46 to 0·62)	20545 (9957-35131)	2·95 (2·58-6)	77049 (36956-130517)	3·5 (3·27-7)	0·46 (0·36 to 0·57)
Low-middle SDI	AF-IS	39589 (21843-67376)	9·89 (5·6-16)	101094 (58289-164910)	9·44 (5·54-15)	-0·26 (-0·31 to -0·21)	578193 (372092-869770)	141·32 (91·54-209)	1412841 (941908-2062028)	130·45 (87·49-188)	-0·28 (-0·33 to -0·23)	35970 (23044-53680)	10·09 (7·08-14)	91712 (61106-132911)	9·34 (6·79-13)	-0·24 (-0·3 to -0·18)
	DM-IS	13525 (5218-27900)	2·71 (1·47-5)	58969 (23891-115555)	4·58 (2·04-8)	1·65 (1·61 to 1·69)	181213 (82963-326245)	37·44 (17·28-66)	748873 (358208-1298506)	59·74 (28·71-102)	1·52 (1·49 to 1·55)	9306 (4251-16741)	2·23 (2·6-4)	38385 (18301-66459)	3·41 (3·18-7)	1·42 (1·37 to 1·46)
Low SDI	AF-IS	12224 (6647-21040)	8·63 (4·83-14)	26649 (15124-44133)	8·01 (4·64-13)	-0·28 (-0·32 to	168767 (101526-275181)	120·53 (73·17-193)	363155 (227332-571692)	110·25 (69·67-170)	-0·29 (-0·34 to	9846 (5851-16076)	8·42 (5·41-13)	22330 (13859-35064)	7·82 (5·29-12)	-0·19 (-0·27 to

						-0·23)					-0·23)					-0·11)
	DM-IS	4746 (1844-9805)	2·66 (1·11-5)	16439 (6613-32579)	3·82 (1·62-7)	1·1 (1·05 to 1·14)	58137 (25128-112352)	34·76 (15·11-66)	195093 (88194-363127)	48·77 (22·22-89)	1·03 (0·97 to 1·09)	2904 (1242-5671)	2·09 (2·32-4)	9694 (4346-18157)	2·83 (2·47-6)	0·98 (0·91 to 1·05)

AF-IS = ischaemic stroke related to atrial fibrillation. ASIR = age-standardised incidence rate. ASMR = age-standardised mortality rate. ASDR = age-standardised DALY rate. AAPC = average annual percentage change. DM-IS = ischaemic stroke related to diabetes mellitus. DALY = disability-adjusted life-year. SDI = sociodemographic index.

Table S4. Differences in global incidence of ischaemic stroke attributable to atrial fibrillation across age and sex in 2021.

Age Group	Sex Group	ASIR (per 100,000 population)	Cases (n)	Lower	Upper
30-34 years	Male	0.004	13	6	26
30-34 years	Female	0.003	8	4	18
35-39 years	Male	0.047	131	55	282
35-39 years	Female	0.029	80	34	168
40-44 years	Male	0.249	627	325	1117
40-44 years	Female	0.133	330	168	606
45-49 years	Male	0.944	2245	1149	4091
45-49 years	Female	0.465	1095	560	2053
50-54 years	Male	2.804	6223	3396	10600
50-54 years	Female	1.293	2882	1607	5055
55-59 years	Male	7.260	14136	7029	26071
55-59 years	Female	3.212	6455	3206	12188
60-64 years	Male	18.083	28125	15773	48948
60-64 years	Female	8.369	13768	7684	24417
65-69 years	Male	43.100	56820	29352	103795
65-69 years	Female	22.481	32373	16675	59598
70-74 years	Male	95.857	92397	52003	153118
70-74 years	Female	51.266	56109	30843	94270

75-79 years	Male	187.954	112371	63789	183511
75-79 years	Female	103.739	74793	41253	127015
80-84 years	Male	284.754	104367	62860	161436
80-84 years	Female	199.694	101706	60002	159786
85-89 years	Male	362.075	62467	40619	91023
85-89 years	Female	331.466	94365	60641	140598
90-94 years	Male	441.104	25709	16329	38173
90-94 years	Female	461.510	55662	35371	84483
95+ years	Male	498.079	7531	4595	11794
95+ years	Female	516.636	20346	12154	31798

ASIR = age-standardised incidence rate.

Table S5. Differences in global mortality of ischaemic stroke attributable to atrial fibrillation across age and sex in 2021.

Age Group	Sex Group	ASMR (per 100,000 population)	Cases (n)	Lower	Upper
30-34 years	Male	0.000	0	0	1
30-34 years	Female	0.000	0	0	0
35-39 years	Male	0.003	8	5	12
35-39 years	Female	0.001	3	2	5
40-44 years	Male	0.018	45	29	67
40-44 years	Female	0.007	17	11	27
45-49 years	Male	0.087	206	134	314
45-49 years	Female	0.031	73	46	115
50-54 years	Male	0.362	804	519	1222
50-54 years	Female	0.120	268	174	409
55-59 years	Male	1.200	2337	1536	3513
55-59 years	Female	0.423	849	545	1308
60-64 years	Male	5.081	7902	5367	11470
60-64 years	Female	1.786	2938	1955	4404
65-69 years	Male	14.327	18887	12848	27583
65-69 years	Female	5.965	8590	5773	12532
70-74 years	Male	43.915	42330	29407	59536
70-74 years	Female	22.765	24915	17161	35045

75-79 years	Male	99.128	59265	41480	81748
75-79 years	Female	59.871	43165	29987	59616
80-84 years	Male	234.327	85885	61322	115480
80-84 years	Female	163.641	83344	56640	113339
85-89 years	Male	479.890	82793	59057	108969
85-89 years	Female	313.405	89223	58557	121767
90-94 years	Male	706.787	41195	28897	53925
90-94 years	Female	530.347	63964	39872	87464
95+ years	Male	717.539	10849	6951	14364
95+ years	Female	739.227	29112	16837	40669

ASMR = age-standardised mortality rate.

Table S6. Differences in global disability-adjusted life years of ischaemic stroke attributable to atrial fibrillation across age and sex in 2021.

Age Group	Sex Group	ASDR (per 100,000 population)	Cases (n)	Lower	Upper
30-34 years	Male	0.023	69	44	106
30-34 years	Female	0.014	42	25	67
35-39 years	Male	0.247	700	450	1072
35-39 years	Female	0.141	390	240	612
40-44 years	Male	1.217	3067	2007	4594
40-44 years	Female	0.623	1546	955	2378
45-49 years	Male	4.964	11808	7726	17631
45-49 years	Female	2.239	5274	3316	8088
50-54 years	Male	17.545	38946	25328	57823
50-54 years	Female	7.074	15771	10103	23772
55-59 years	Male	50.557	98447	64728	144159
55-59 years	Female	20.689	41585	26994	63203
60-64 years	Male	171.805	267220	182073	382239
60-64 years	Female	66.996	110216	72486	163814
65-69 years	Male	406.855	536370	367899	765290
65-69 years	Female	182.799	263246	176729	381817
70-74 years	Male	1002.736	966551	673670	1351910
70-74 years	Female	535.776	586394	404688	821617

75-79 years	Male	1818.401	1087162	767930	1486019
75-79 years	Female	1103.349	795488	554425	1098642
80-84 years	Male	3300.884	1209835	872772	1613056
80-84 years	Female	2277.097	1159752	798294	1573221
85-89 years	Male	5199.189	896998	644705	1168041
85-89 years	Female	3431.314	976865	648685	1327582
90-94 years	Male	6567.885	382809	271350	498834
90-94 years	Female	4967.724	599150	382673	815843
95+ years	Male	6318.838	95543	63144	126103
95+ years	Female	6402.618	252152	150853	350671

ASDR = age-standardised disability-adjusted life years rate.

Table S7. Differences in global incidence of ischaemic stroke attributable to diabetes across age and sex in 2021.

Age Group	Sex Group	ASIR (per 100,000 population)	Cases (n)	Lower	Upper
30-34 years	Male	0.228	695	244	1574
30-34 years	Female	0.208	620	211	1441
35-39 years	Male	0.448	1268	393	3088
35-39 years	Female	0.381	1057	331	2519
40-44 years	Male	1.089	2745	1060	5539
40-44 years	Female	0.793	1966	745	4088
45-49 years	Male	2.508	5966	2275	12286
45-49 years	Female	1.649	3884	1478	8243
50-54 years	Male	5.048	11206	4562	21558
50-54 years	Female	3.165	7056	2934	13983
55-59 years	Male	9.155	17827	6610	37165
55-59 years	Female	5.641	11338	4205	24154
60-64 years	Male	16.843	26196	10935	51687
60-64 years	Female	10.778	17730	7390	35487
65-69 years	Male	29.285	38607	14758	80547
65-69 years	Female	19.602	28228	10813	58981
70-74 years	Male	48.828	47065	19417	90096
70-74 years	Female	31.019	33950	13764	65400

75-79 years	Male	73.519	43954	18054	84144
75-79 years	Female	45.246	32621	13110	64418
80-84 years	Male	86.009	31523	13524	58147
80-84 years	Female	64.193	32694	13844	60735
85-89 years	Male	90.979	15696	7176	27652
85-89 years	Female	85.023	24205	11019	43253
90-94 years	Male	96.283	5611	2481	10177
90-94 years	Female	99.550	12006	5353	22080
95+ years	Male	101.923	1541	653	2955
95+ years	Female	103.327	4069	1703	7714

ASIR = age-standardised incidence rate.

Table S8. Differences in global mortality of ischaemic stroke attributable to diabetes across age and sex in 2021.

Age Group	Sex Group	ASMR (per 100,000 population)	Cases	Lower	Upper
30-34 years	Male	0.012	36	17	63
30-34 years	Female	0.007	20	9	38
35-39 years	Male	0.029	82	39	140
35-39 years	Female	0.016	45	21	81
40-44 years	Male	0.078	197	94	337
40-44 years	Female	0.042	103	48	185
45-49 years	Male	0.231	549	265	943
45-49 years	Female	0.110	260	123	462
50-54 years	Male	0.652	1447	697	2485
50-54 years	Female	0.294	656	319	1132
55-59 years	Male	1.514	2947	1445	5009
55-59 years	Female	0.742	1492	715	2593
60-64 years	Male	4.732	7360	3721	12112
60-64 years	Female	2.300	3783	1880	6401
65-69 years	Male	9.734	12833	6460	21405
65-69 years	Female	5.202	7490	3744	12402
70-74 years	Male	22.370	21562	10980	35031
70-74 years	Female	13.774	15075	7658	24313

75-79 years	Male	38.774	23181	11740	37483
75-79 years	Female	26.113	18826	9530	30235
80-84 years	Male	70.778	25941	13193	41594
80-84 years	Female	52.604	26791	13069	43080
85-89 years	Male	120.583	20803	10434	33104
85-89 years	Female	80.390	22886	10641	37460
90-94 years	Male	154.276	8991	4391	14376
90-94 years	Female	114.398	13797	6035	22859
95+ years	Male	146.831	2220	988	3599
95+ years	Female	147.845	5822	2360	9867

ASMR = age-standardised mortality rate.

Table S9. Differences in global disability-adjusted life years of ischaemic stroke attributable to diabetes across age and sex in 2021.

Age Group	Sex Group	ASDR (per 100,000 population)	Cases	Lower	Upper
30-34 years	Male	1.161	3548	1676	6200
30-34 years	Female	0.991	2963	1318	5412
35-39 years	Male	2.382	6742	3221	11711
35-39 years	Female	1.848	5134	2341	9136
40-44 years	Male	5.327	13432	6536	22781
40-44 years	Female	3.704	9190	4218	16030
45-49 years	Male	13.189	31371	15292	52951
45-49 years	Female	7.940	18710	8753	32470
50-54 years	Male	31.590	70124	34015	117589
50-54 years	Female	17.321	38615	18443	65749
55-59 years	Male	63.756	124148	60870	205503
55-59 years	Female	36.339	73041	35402	125250
60-64 years	Male	160.022	248893	126228	403626
60-64 years	Female	86.274	141929	69716	238089
65-69 years	Male	276.441	364441	184981	593879
65-69 years	Female	159.392	229538	114606	377865
70-74 years	Male	510.773	492340	251536	795480
70-74 years	Female	324.181	354808	180595	569997

75-79 years	Male	711.275	425247	217352	681373
75-79 years	Female	481.232	346957	176197	557195
80-84 years	Male	997.017	365425	187776	580999
80-84 years	Female	731.993	372813	184197	597986
85-89 years	Male	1306.410	225390	113904	354841
85-89 years	Female	880.152	250571	117881	408414
90-94 years	Male	1433.622	83558	41240	132993
90-94 years	Female	1071.560	129239	57920	213232
95+ years	Male	1293.031	19551	8975	31596
95+ years	Female	1280.517	50430	21147	85080

ASDR = age-standardised disability-adjusted life years rate.

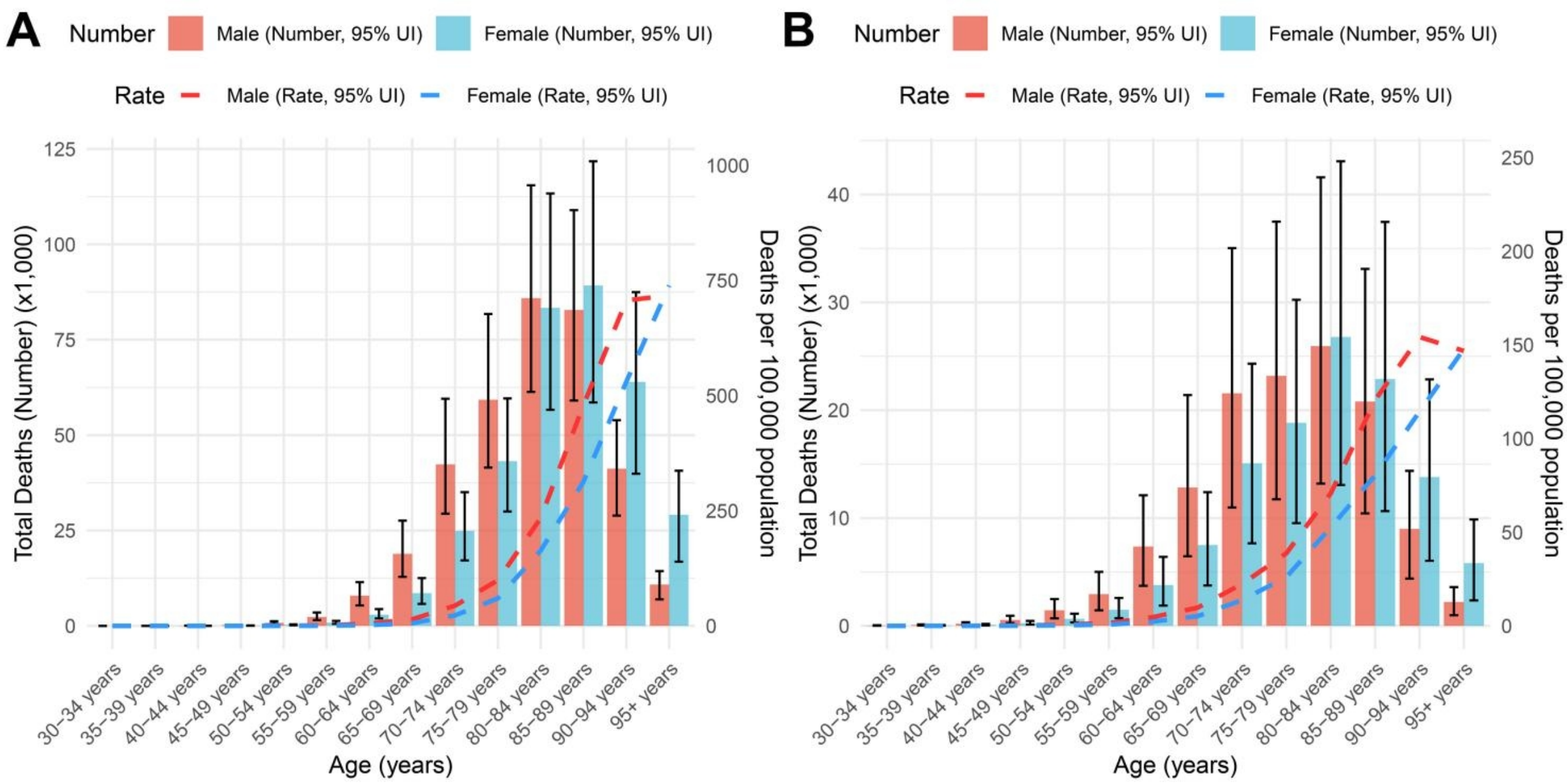

Figure S1. Number of deaths and age-standardized mortality rates for ischaemic stroke attributable to atrial fibrillation (A) and diabetes (B) by sex in 2021.

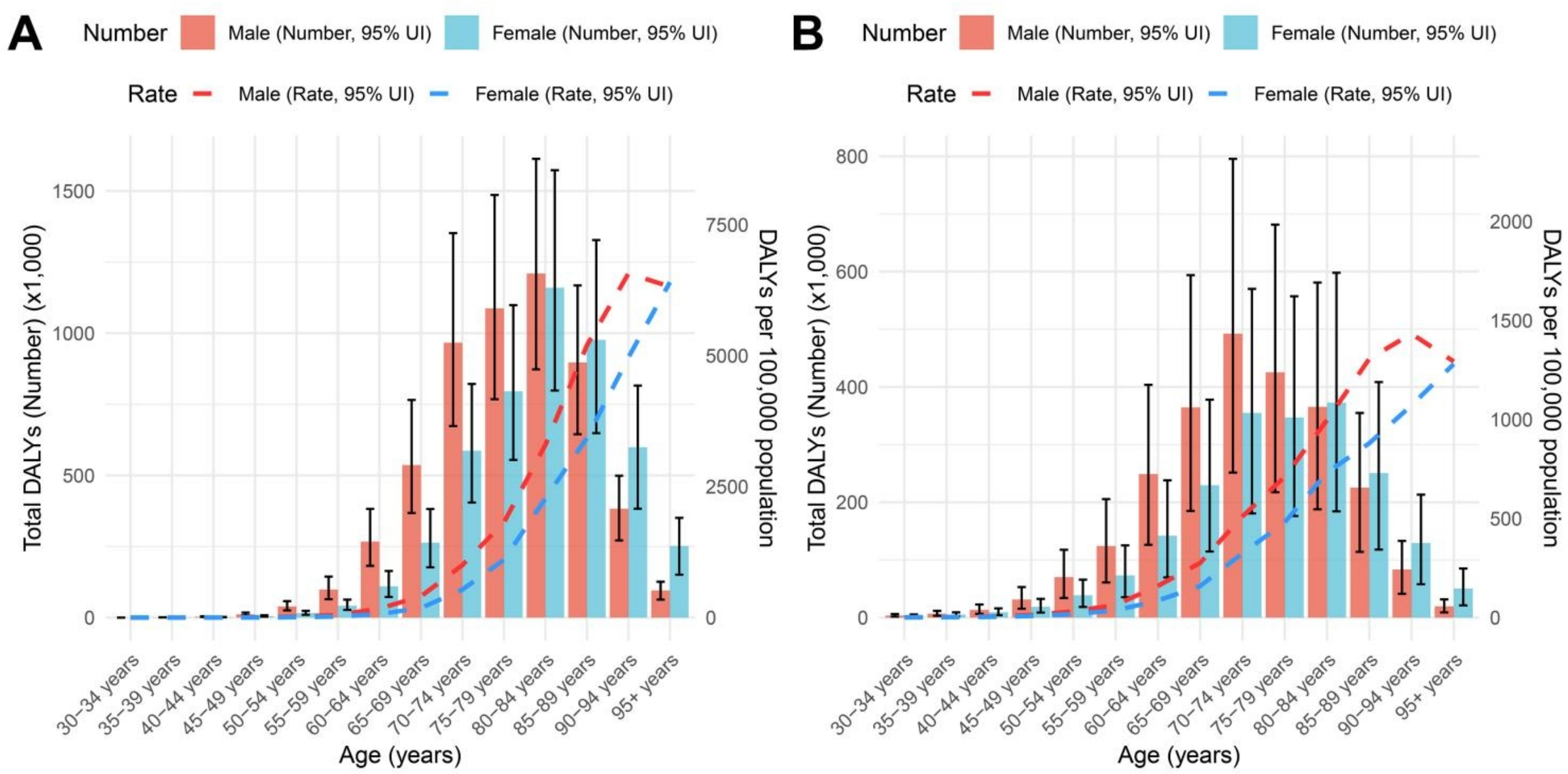

Figure S2. Number of disability-adjusted life years and age-standardized disability-adjusted life year rates for ischaemic stroke attributable to atrial fibrillation (A) and diabetes (B) by sex in 2021.

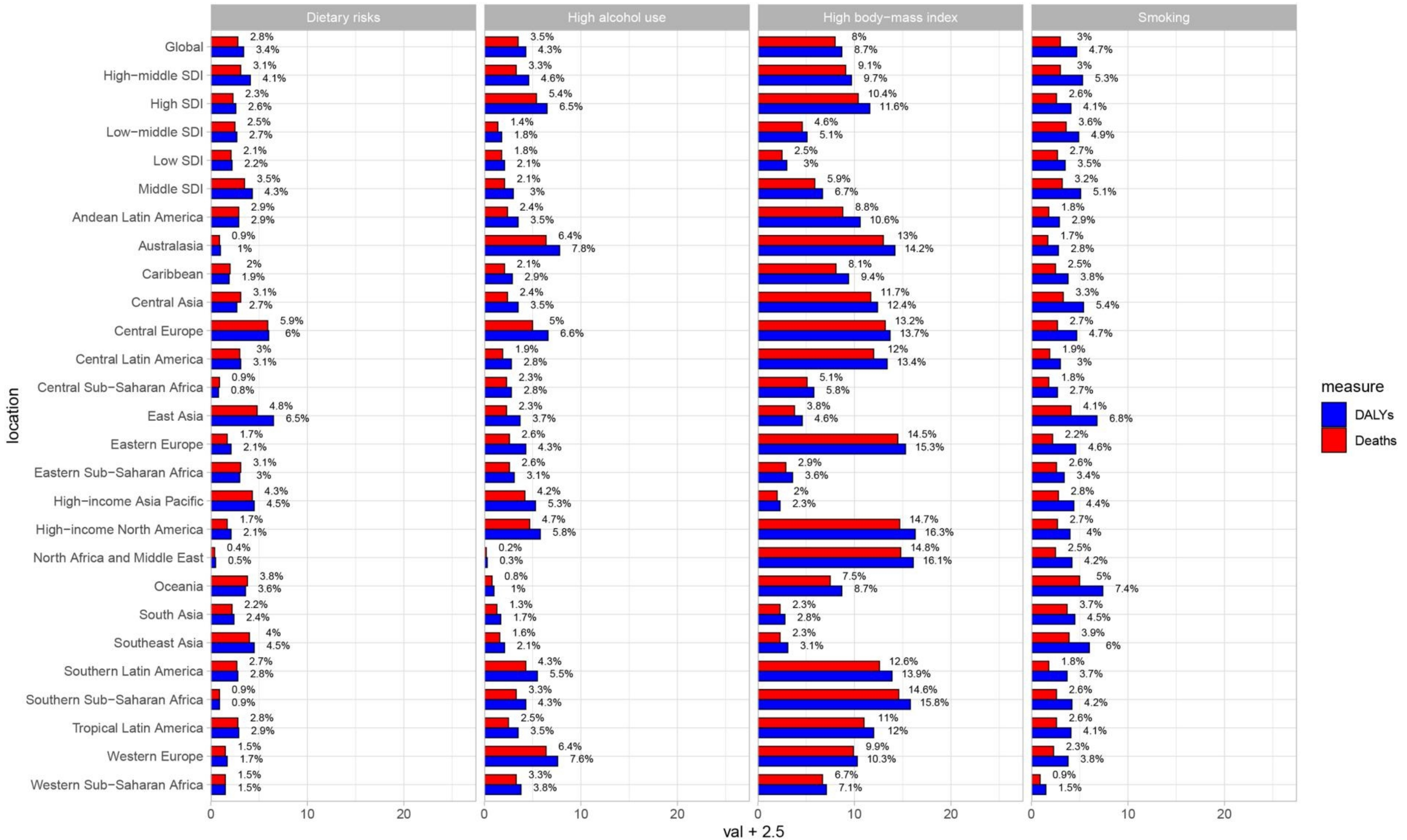

Figure S3. Proportion attributable fraction of atrial fibrillation due to high body mass index, smoking, high alcohol consumption, and dietary risks across regions in 2021.

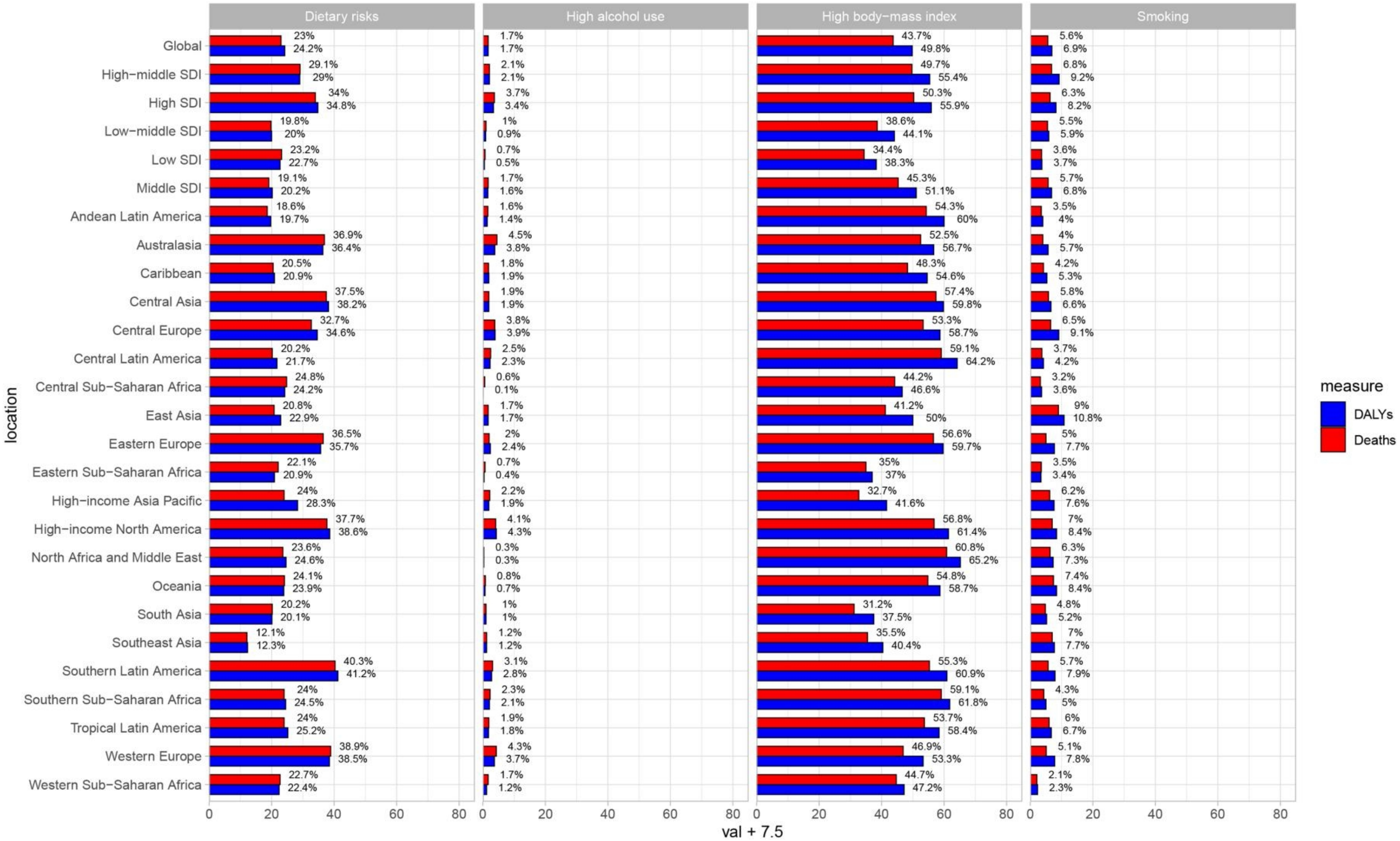

Figure S4. Proportion attributable fraction of diabetes due to high body mass index, smoking, high alcohol consumption, and dietary risks across regions in 2021.

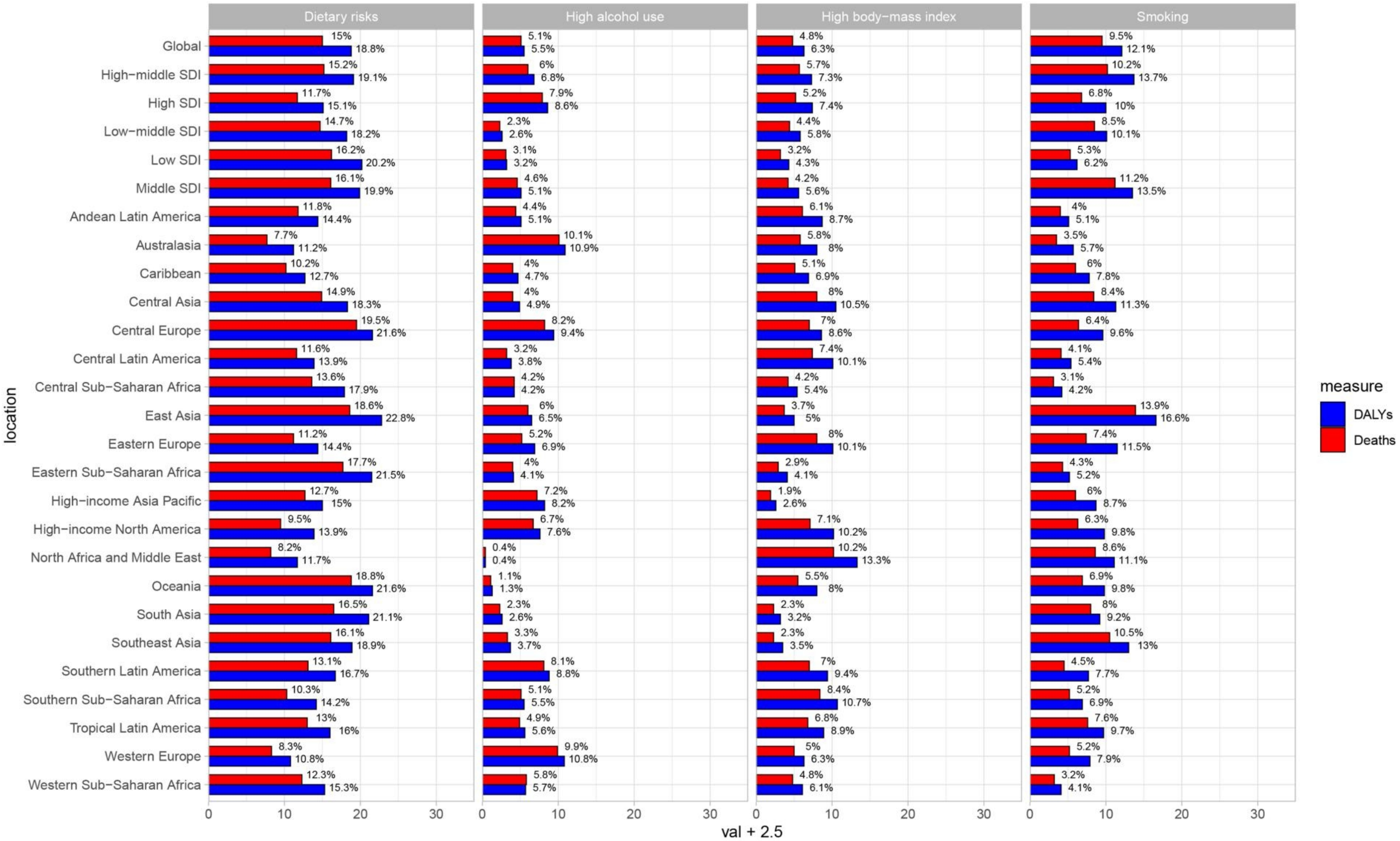

Figure S5. Proportion attributable fraction of ischaemic stroke due to high body mass index, smoking, high alcohol consumption, and dietary risks across regions in 2021.
